# Calcium Indicators with Fluorescence Lifetime-Based Signal Readout: A Structure–Function Study

**DOI:** 10.3390/ijms252312493

**Published:** 2024-11-21

**Authors:** Tatiana R. Simonyan, Larisa A. Varfolomeeva, Anastasia V. Mamontova, Alexey A. Kotlobay, Andrey Y. Gorokhovatsky, Alexey M. Bogdanov, Konstantin M. Boyko

**Affiliations:** 1 Shemyakin-Ovchinnikov Institute of Bioorganic Chemistry, Moscow 117997, Russia; tatiana.simonyan7@gmail.com (T.R.S.); sphingozin@gmail.com (A.V.M.); andrey.gorokhovatsky@yandex.ru (A.Y.G.); 2 A.N. Bach Institute of Biochemistry, Research Centre of Biotechnology of the Russian Academy of Sciences, Moscow 119071, Russia; l.varfolomeeva@fbras.ru; 3 Department of Photonics, İzmir Institute of Technology, 35430 İzmir, Turkey

**Keywords:** quantitative calcium imaging, FLIM, fluorescence lifetime, genetically encoded indicators, structural analysis

## Abstract

The calcium cation is a crucial signaling molecule involved in numerous cellular pathways. Beyond its role as a messenger or modulator in intracellular cascades, calcium’s function in excitable cells, including nerve impulse transmission, is remarkable. The central role of calcium in nervous activity has driven the rapid development of fluorescent techniques for monitoring this cation in living cells. Specifically, genetically encoded calcium indicators (GECIs) are the most in-demand molecular tools in their class. In this work, we address two issues of calcium imaging by designing indicators based on the successful GCaMP6 backbone and the fluorescent protein BrUSLEE. The first indicator variant (GCaMP6s-BrUS), with a reduced, calcium-insensitive fluorescence lifetime, has potential in monitoring calcium dynamics with a high temporal resolution in combination with advanced microscopy techniques, such as light beads microscopy, where the fluorescence lifetime limits acquisition speed. Conversely, the second variant (GCaMP6s-BrUS-145), with a flexible, calcium-sensitive fluorescence lifetime, is relevant for static measurements, particularly for determining absolute calcium concentration values using fluorescence lifetime imaging microscopy (FLIM). To identify the structural determinants of calcium sensitivity in these indicator variants, we determine their spatial structures. A comparative structural analysis allowed the optimization of the GCaMP6s-BrUS construct, resulting in an indicator variant combining calcium-sensitive behavior in the time domain and enhanced molecular brightness. Our data may serve as a starting point for further engineering efforts towards improved GECI variants with fine-tuned fluorescence lifetimes.

## 1. Introduction

To date, researchers have access to a broad repertoire of approaches for visualizing calcium cations and calcium transients in biological systems. These include both label-free techniques, such as measurements using a plasmonic-based electrochemical impedance microscope (P-EIM) [1], and the luminescent detection of signals from molecular calcium indicators. Among the latter, a group of small synthetic dyes, such as Fura-2, and Fluo-3 [2], which have become the ‘living classics’ of calcium imaging, and several families of genetically encoded calcium indicators (GECIs) based on GFP-like fluorescent proteins [3,4,5,6], bilirubin-binding bacterial phytochromes [7,8], and calcium-sensitive bioluminescent systems [9,10], can be highlighted.

Calcium imaging based on genetically encoded fluorescent indicators (GECIs) has become one of the most important sensing techniques for neurobiology over the past decade. State-of-the-art GECIs are employed for the indirect detection of electrical activity down to subthreshold events [11,12] with a cellular or even subcellular spatial resolution [13,14,15] on a time scale of down to milliseconds [5], across a broad spectrum of studies dedicated to all levels of the nervous system organization in higher multicellular organisms [3,4,6,16,17,18]. Although calcium indicators are represented by a rich palette of both intensiometric (i.e., monocolored, single fluorophore) [7,19,20,21,22] and ratiometric (bearing two fluorophores) [8,23,24,25,26,27] variants, several families of the former group (especially GECO [28,29] and GCaMP [5,30,31]) have become most in demand in in vivo studies due to their comparative technical simplicity of visualization and good kinetic response characteristics. Constant efforts are being made to increase the molecular brightness [27,32,33], dynamic range [33,34,35], and kinetic rate [5,35,36] of calcium indicators by replacing and/or optimizing the fluorescent protein-based reporter domain [33,35], changing and/or optimizing the topology of the chimeric molecule [35], and modifying the calcium-sensing domain of the indicator [5,27,36].

Despite the advantages of state-of-the-art GECIs, most of them are not adapted to solving some specialized tasks of calcium imaging. For example, due to the influence of many experimental factors on fluorescence intensity, the signal of intensiometric indicators is barely suitable for the quantitative analysis of calcium levels [37,38], and ratiometric indicators can be used to measure the absolute concentration of the analyte with many reservations [39,40]. As a result, steady-state measurements of calcium homeostasis, as well as the assessment of absolute changes in calcium concentration during signaling, remain challenging. Using the fluorescence lifetime instead of intensity could greatly facilitate the quantitative analysis of the indicator signal, but most GECIs exhibit lifetimes that are almost insensitive to calcium fluctuations [38,41]. The few exceptions, such as RCaMP [40], show a rather modest overall lifetime change contrast, which is typically less than 1 ns [38,40]. Although efforts in designing new indicators specifically targeted at increasing the sensitivity of their fluorescence lifetime to calcium have been quite successful—in the case of the Tq-Ca-FLITS indicator, a threefold change in lifetime for a total of 1.3 ns was achieved [38]—the number of such variants is small, and their spectral properties are suboptimal for live-cell or in vivo imaging. Thus, in our opinion, there remains a relevant request from the scientific community for the development of new indicators of this type.

Another, less obvious field where the fluorescence lifetime acts as a critical parameter is the family of imaging modalities, where multiple axially spaced and time-shifted focal areas capture the full axial imaging range almost simultaneously, thus enabling mesoscale and volumetric imaging with an unprecedentedly high temporal resolution [42,43]. Since acquisition speed in the case of light beads microscopy (LBM) and similar techniques is limited only by the fluorophore’s lifetime, calcium indicators with shortened lifetimes would contribute to a proportional increase in imaging speed (and, thus, temporal resolution). In this case, however, the pronounced sensitivity of the fluorescence lifetime to calcium is a disadvantage, as it does not allow standardizing the hardware settings for signal collection.

In this study, we address both aforementioned fluorescence lifetime-related issues of calcium imaging by designing indicators based on the GCaMP6 backbone and the fluorescent protein BrUSLEE. The latter is a close homolog of the widely used EGFP protein [44], whose circular permutant constitutes the fluorescent core of the original GCaMP6 [19]. BrUSLEE exhibits a sub-nanosecond fluorescence lifetime with an acceptable molecular brightness [45,46]. Moreover, amino acid substitutions in the chromophore-forming triad and the nearest chromophore environment apparently provide the chromophore of this protein with greater conformational mobility [46,47] (and, hence, flexibility of fluorescence lifetime) compared to the parental EGFP. Importantly, similar to the parental protein, BrUSLEE tolerates circular permutation [48]. Taken together, the properties of BrUSLEE create prerequisites for solving the specific tasks of calcium imaging mentioned above.

## 2. Results and Discussion

### 2.1. Genetic Constructs

**Figure 1:**
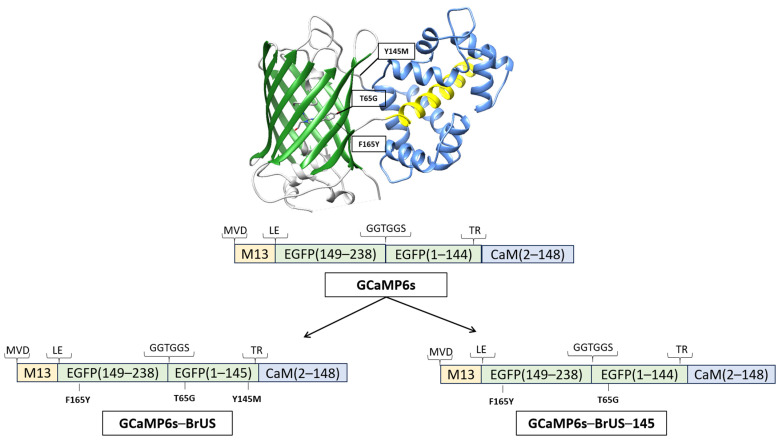
Schematics showing the design of chimeric proteins used in the study. At the top, the spatial structure of GCaMP6 is shown, with the mutations required for the EGFP modification. In the center, there is a linear scheme of the GCaMP-type backbone. The numbering of amino acid positions corresponds to those of the unmodified proteins (EGFP and calmodulin). At the bottom (left and right), the modified variants, GCaMP6s-BrUS and GCaMP6s-BrUS-145, are displayed, with the introduced modifications displayed below them.

At the first stage of this study, we replaced circularly permuted EGFP (cpEGFP)—the fluorescent core of GCaMP6s—with circularly permuted BrUSLEE. Since the permutation procedure in the original GCaMP6s involves the deletion of a 4-amino-acid fragment (corresponding to positions 145–148 in the parental protein) [19], and, thus, the removal of the 145th amino acid residue, which is an important structural determinant of the fluorescent properties of BrUSLEE [45,46], we engineered two variants of the construct, with (GCaMP6s-BrUS) and without (GCaMP6s-BrUS-145) the M145 residue (Figure 1).

### 2.2. Spectral Properties and Calcium Sensitivity

The obtained constructs, along with the plasmid encoding the parental GCaMP6s, were expressed in a bacterial system. In bacterial cells, cpBrUSLEE-based chimeric proteins showed a fluorescence signal appearance time comparable to that of GCaMP6s (this value estimates the apparent chromophore maturation rate), but with a slightly reduced average cellular fluorescence brightness. The proteins were then extracted, purified, and subjected to steady-state spectroscopy measurements. Both their absorption and fluorescence properties were found to be similar to each other and to the parental protein, with just a slight hypsochromic shift in their excitation maxima (Supplementary Figure S1).

Under conditions of calcium indicator molecule saturation (Sat-form, 39 μM calcium), GCaMP6s-BrUS showed a molecular brightness (EC × FQY) circa three-fold lower, albeit still comparable to that of the original GCaMP6s. In contrast, the GCaMP6s-BrUS-145 variant was found to be extremely dim, showing only 10.5% of GCaMP6s’ brightness (Table 1). In a calcium-free solution, all three proteins (Apo-form) showed a drastic drop in their brightness, as expected. A decrease in the extinction coefficient was an important reason for the brightness shift in all indicator variants. Conversely, the contribution of the fluorescence quantum yield to the calcium sensitivity differed significantly among them. Specifically, GCaMP6s showed only a slight reduction in quantum yield in the calcium-free environment, whereas both GCaMP6s-BrUS and GCaMP6s-BrUS-145 displayed a well-marked decrease in their quantum yields, especially pronounced (five-fold) for the latter variant (Table 1). It should also be noted that the results of our measurements of the molecular brightness of GCaMP6s do not perfectly match those shown in the original publication; this discrepancy may be due to differences in measurement methodology and conditions.

**Figure 2:**
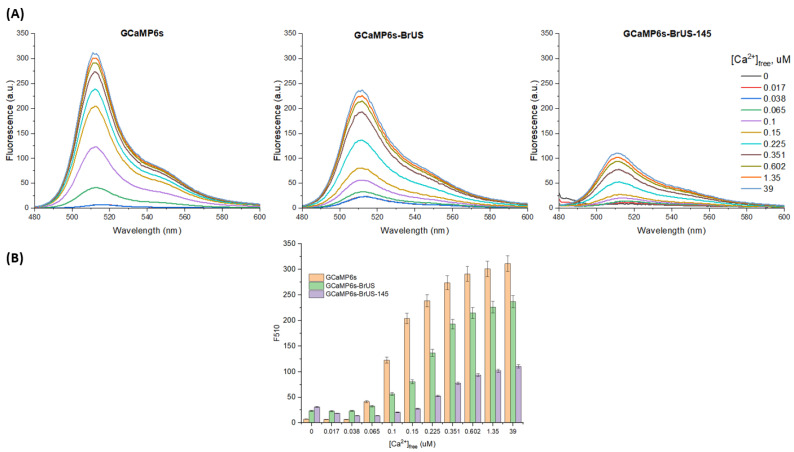
Calcium sensitivity of purified GCaMP6s, GCaMP6s-BrUS, and GCaMP6s-BrUS-145 measured in the intensiometric mode. (**A**) The dependence of fluorescence intensity at 510 nm (λ_ex_ = 475 nm) on calcium concentration, expressed as [Ca^2+^]_free_ (see Supplementary Table S1 for details on the correspondence between [Ca^2+^]_free_ and [CaEGTA]). (**B**) Column histogram displaying the relative fluorescence intensity changes observed within the [Ca^2+^]_free_ range of 0–39 μM (corresponds to the [CaEGTA] range of 0–10 mM). Standard errors of the mean (S.E.M.) are shown for each data point (*n* = 3).

Next, we determined the dynamic range of the indicators in vitro by measuring the dependence of the fluorescence intensity of the purified proteins on the calcium concentration (Figure 2). The original GCaMP6s variant demonstrated the highest dynamic range, with approximately a 40-fold change in the amplitude of the fluorescence emission spectrum at its peak when the free calcium concentration in the sample increased from 0 to 39 µM. In contrast, both cpBrUSLEE-based variants showed a noticeable relative decrease in the dynamic range. For both GCaMP6s-BrUS and GCaMP6s-BrUS-145, the dynamic range was approximately 10. Additionally, GCaMP6s-BrUS-145 exhibited some narrowing of the working range, as the indicator readings for free calcium concentrations below 150 nM (5 mM [CaEGTA]) were barely distinguishable.

**Figure 3:**
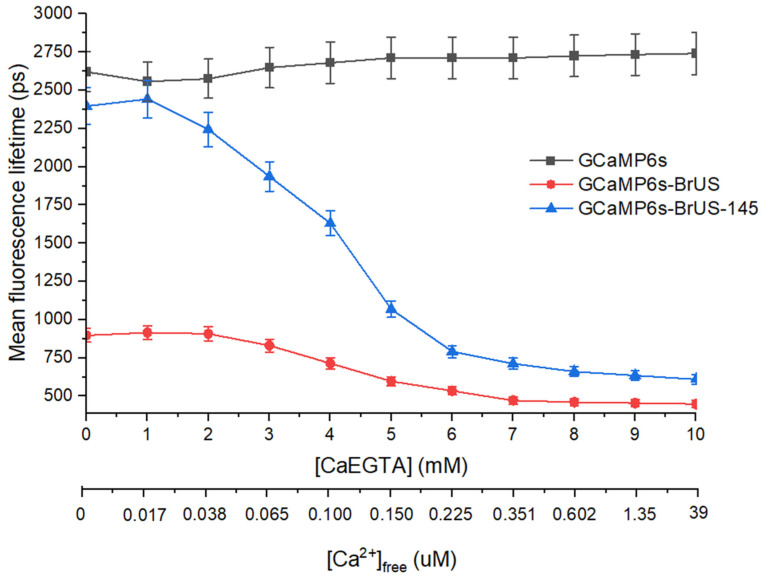
The graph describing the dependence of the amplitude-weighted mean fluorescence lifetime of indicator variants on calcium concentration ([CaEGTA] and [Ca^2+^]_free_; see Supplementary Table S1 for details). λ_ex_ = 450 nm, repetition rate is 20 MHz. Standard errors of the mean (S.E.M.) are shown for each data point (*n* = 3).

To evaluate the sensitivity of the fluorescence lifetime of indicators to calcium, we measured the kinetics of their fluorescence decay using single-photon excitation with a picosecond laser emitting at a wavelength of 450 nm, under consistent conditions. The fluorescence decay of the parental GCaMP6s was adequately fitted by a mono-exponential function with a lifetime of approximately 2.6 ns (Supplementary Figure S2), which is weakly dependent on the calcium concentration (Figure 3). Both cpBrUSLEE-based variants exhibited multi-component decay kinetics, best fitted by a tri-exponential model. The individual exponential decay components of GCaMP6s-BrUS and GCaMP6s-BrUS-145 displayed some calcium-dependent behavior (Supplementary Figures S3 and S4); however, they do not form a monotone dependence and are thus difficult to use as an indicator readout. Only the long-lived component (τ_3_) of both proteins showed a clear tendency to shorten its lifetime value and relative contribution (amplitude, A3) with an increasing calcium concentration (Supplementary Figures S3 and S4). The amplitude-weighted mean fluorescence lifetime (τ_m_) turned out to be a more informative parameter (Figure 3). For GCaMP6s-BrUS, it appeared to be weakly sensitive to the analyte concentration, slightly decreasing (from approximately 750 to 500 ps) with the calcium concentration increasing from 38 to 350 nM (2–7 mM [CaEGTA]). The mean lifetime of GCaMP6s-BrUS-145 exhibited a well-defined dependence on calcium concentration: as the calcium concentration increases from 17 to 350 nM (1–7 mM [CaEGTA]), it decreases from approximately 2.4 ns to 0.8 ns. Similarly to the case of intensiometry, the signal value for the points 0 and 17 nM does not show significant differences (Figure 3).

Overall, our initial assumptions that using cpBrUSLEE instead of cpEGFP as the fluorescent core of the calcium indicator could provide a shorter fluorescence lifetime and/or increased lifetime sensitivity to calcium were experimentally confirmed. Indeed, the GCaMP6s-BrUS variant, while maintaining a relatively high molecular brightness, demonstrated an extremely short mean fluorescence lifetime with a low sensitivity to calcium. In contrast, the GCaMP6s-BrUS-145 variant exhibited a pronounced calcium-dependent change in its mean lifetime over a wide range. However, the structural determinants of the photobehavior features of both indicator variants, and thus the possibility of their further rational modification, remained unclear. This mainly included the nature of the differences between GCaMP6s-BrUS and GCaMP6s-BrUS-145, differing by one amino acid in the chain. Moreover, an important drawback of GCaMP-BrUS-145 is its extremely low molecular brightness (Table 1), which may hinder its practical application in living cells due to the potential influence of fluorescence from endogenous intracellular fluorophores (so-called autofluorescence) and the high dose of absorbed energy required to accumulate a sufficient number of photons when measuring the indicator signal. Another notable drawback of GCaMP-BrUSLEE-145 is its reduced sensitivity to calcium in the nanomolar (up to tens of nanomoles) range.

Given these circumstances, we decided to study the structure of both indicator variants to gain structural insights into the molecular bases of their calcium sensitivity and/or fluorescence properties.

### 2.3. Crystal Structure of GCaMP6s-BrUS and GCaMP6s-BrUS-145

Recombinant proteins GCaMP6s-BrUS and GCaMP6s-BrUS-145 were isolated and purified through a series of chromatography steps, including ion exchange (Supplementary Figures S5–S8), size-exclusion (Supplementary Figures S9–S12), and ultrafiltration procedures. The purity of the resulting samples was assessed using SDS-PAGE (Supplementary Figure S13). Subsequently, the purified GCaMP6s-BrUS and GCaMP6s-BrUS-145 samples were utilized for crystallization.

**Figure 4:**
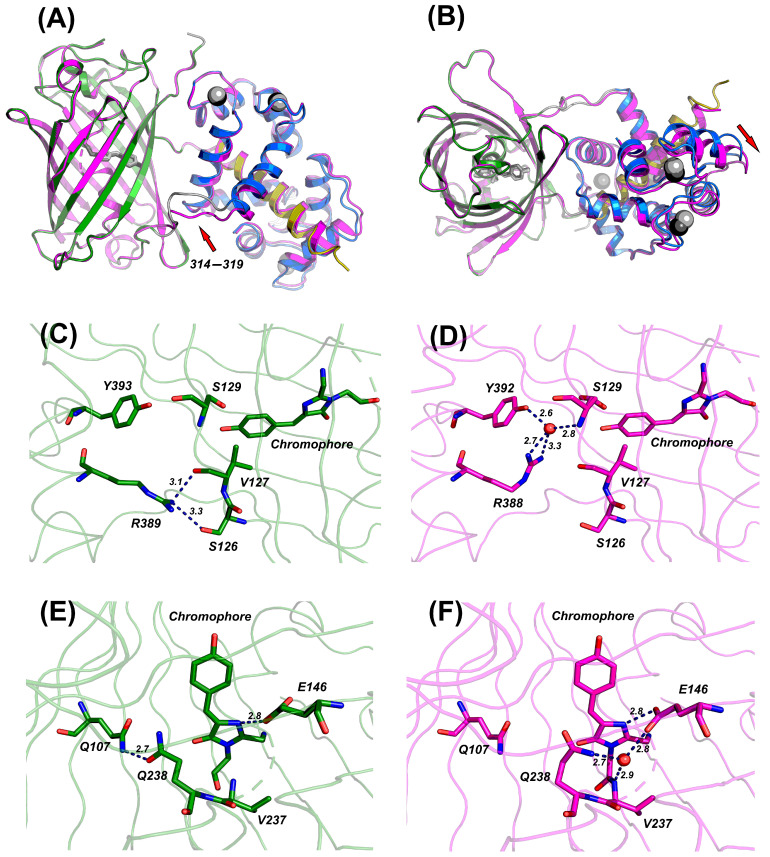
Comparison of GCaMP6s-BrUS and GCaMP6s-BrUS-145 structures. (**A**,**B**) Superposition of GCaMP6s-BrUS and GCaMP6s-BrUS-145 (magenta) structures from two views. Color scheme for GCaMP6s-BrUS is the following: EFGP domain—green, CaM domain—blue, M13 helix—orange, and the linker 314–319—light gray. Chromophore and calcium ions are shown in light and dark gray color for GCaMP6s-BrUS and GCaMP6s-BrUS-145, respectively. Red arrows point to the shift of the linker 314–319 (**A**) and the C-lobe of the CaM domain (**B**). (**C**–**F**) Differences in the conformation of residues surrounding the chromophore. Panels (**C**,**E**) represent GCaMP6s-BrUS structure and (**D**,**F**) GCaMP6s-BrUS-145. Solvent molecules are shown as red spheres. Hydrogen bonds are depicted as dashed blue lines.

Both proteins crystallized in the same space group and have a similar overall architecture, which superposed with the RMSD value of about 0.7 Å (by all Cα atoms), among which the GFP domains of both indicators (residues 72–313) have a very low corresponding RMSD value of 0.3 Å indicating the overall stability of the domain, while, for the CaM domains, this value reaches 0.5 Å (Figure 4A,B). The most remarkable differences were observed in the following regions: the mobile linker ^314^(-/M)LPDQL^319^, together with the part of the first α-helix of the CaM domain, where the distance between the correspondent Cα atoms reaches 4.4 Å and the last Ca-binding site at residues 428–443 (2.4 Å)—other minor discrepancy was found in the 320–324 region. It is noteworthy that the shortening of the 314–319 linker led to a significant and simultaneous shift of this linker as well as the C-lobe of the CaM domain and M13 helix compared to the GCaMP6s-BrUS structure, reflecting the increased rigidity of the shortened linker.

In both structures, mature chromophores formed by the tripeptide GYG are covalently bound to the polypeptide chain and have a clear electron density. Four calcium ions are tightly bound in CaM domains at the corresponding binding sites, confirming the holo-form of the indicator. In both indicator variants, chromophores are in a very similar trans-conformation. In GCaMP6s-BrUS, the phenol moiety of the chromophore is surrounded by a mixture of hydrophobic (V74, I91, V127, and V230) and polar residues (Y89, S129, E146, and T231). Some of these residues participate in the chromophore fixation via a network of hydrogen bonds, including E146 and V230, as well as Q263, R265, and solvent-mediated Y261 and Q238. Compared to GCaMP6s-BrUS, the resolution of GCaMP6s-BrUS-145 is higher, allowing the visualization of extra two solvent molecules in the vicinity of the chromophore. These molecules mediate the hydrogen bonding of the OH-atom of the tyrosine moiety of the chromophore with the side-chain of S129 as well as the main chain oxygens of E72 and H90 and the main chain nitrogen of V127.

A comparison of the chromophore environment in both structures allowed us to identify amino acid residues that change their conformation, which might affect the fluorescence properties of the chromophore (Figure 4C–F). One is arginine R389, which exhibits different conformations of its side chain in both structures. This residue together with R67, E72, Q73, S126, Q128, T385, A372, and Y393 participates in the formation of the side wall of the protein shielding the chromophore. In GCaMP6s-BrUS, R389 interacts with the side chain of S126 and the main chain oxygen of V127 (Figure 4C). In contrast, in the GCaMP6s-BrUS-145 structure, the guanidine group of the corresponding R388 is turned to the opposite side in the direction of the mobile linker ^315^LPDQL^319^ and forms the solvent-mediated hydrogen bond with the side chain oxygen of Y392 and the main chain nitrogen of S129 (Figure 4D). Such changes in the conformation of R389 (R388) may influence the solvent accessibility of the chromophore, potentially controlling the degree of solvent-based fluorescence quenching. This, in turn, might affect both the fluorescence brightness and lifetime flexibility in this sensor variant.

Another residue, whose side chain conformation is different in both structures, is E146 (Figure 4E,F). This residue is located near the chromophore and fixes its imidazolic moiety via a hydrogen bond to the N2 atom; however, the conformation of its side chain differs, which might also affect the properties of the chromophore. It is noteworthy that, in the GCaMP6s-BrUS-145 structure, the side chain of E146 forms an extra water-mediated hydrogen bond to the main chain nitrogen of V237 and the side chain of Q238 (Figure 4F). The latter, being located in the vicinity of the chromophore, is the third residue, which changed its conformation upon the mutation being introduced. In case of GCaMP6s-BrUS, the side chain of Q238 is oriented off the E146 and forms a tight hydrogen bond with the side chain of Q107 (Figure 4E). The corresponding bond is absent in the case of GCaMP6s-BrUS-145.

Based on the structural data for the two indicator variants, the following modifications were proposed for three amino acid positions of GCaMP6s-BrUS: M314 and D317 (in the interdomain linker region), and R389 (in the fluorescence core of the indicator). Our rationale for choosing these positions includes the following points:

1. The fluorescence lifetime sensitivity of the circularly permuted BrUSLEE to the analyte may be determined by the rigidity of its connection to the calcium-sensitive domain (up to the possibility of some conformational twisting of the chromophore in response to calcium binding). Since our X-ray data revealed a well-defined difference in the linker configurations between GCaMP6s-BrUS and GCaMP6s-BrUS-145, we proposed the following:
  (a) The neutralization of the possible contribution of the long-chain polar residue M314 by replacing it with a glycine residue;
  (b) The alteration of the linker mobility by replacing the long-chain charged residue D317 with glycine or serine, which are commonly used to adjust linker flexibility in chimeric proteins.
2. The key role in the conformationally dependent changes in the fluorescence lifetime of the BrUSLEE chromophore may be played by the (variable) solvent accessibility of the chromophore. In GCaMP6s-BrUS, it is primarily controlled by the R389 residue. We propose to replace the large side chain of arginine with three alternative substitutes: glycine, to radically disrupt this chromophore-protein wall interface region; threonine, a medium-sized polar residue capable of making this region more solvent-accessible while retaining some “protection” of the chromophore; and lysine, a long-chain charged residue that can sterically act similarly to the original arginine but with a higher chain mobility and fewer valencies for hydrogen bond formation.

### 2.4. Structure-Guided Mutants, Their Properties, and Further Modification

**Figure 5:**
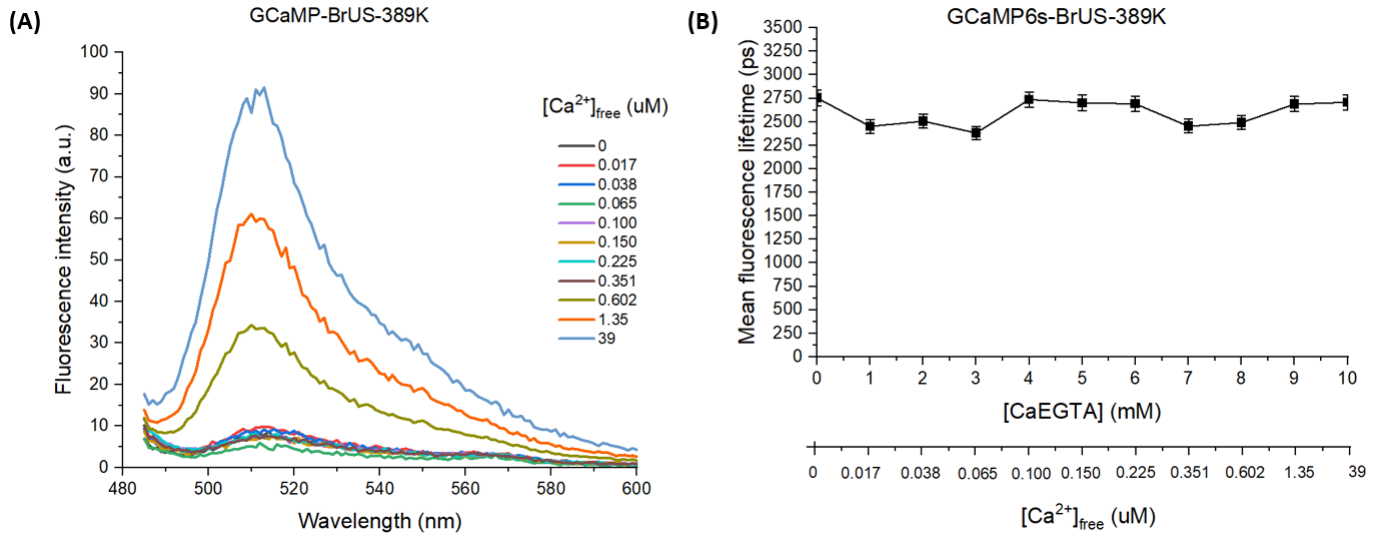
Calcium sensitivity of GCaMP6s-BrUS-389K fluorescence. (**A**) The dependence of fluorescence intensity at 510 nm (λ_ex_ = 475 nm) on calcium concentration, expressed as [Ca^2+^]_free_. (**B**) The graph describing the dependence of the amplitude-weighted mean fluorescence lifetime on calcium concentration ([CaEGTA] and [Ca^2+^]_free_). λ_ex_ = 450 nm, repetition rate is 20 MHz. Standard errors of the mean (S.E.M.) are shown for each data point (*n* = 3).

Using site-directed mutagenesis, the following point mutants were engineered: M314G, D317S, D317G, R389G, R389T, and R389K. All of them were then expressed in a bacterial system, but only the GCaMP6s-BrUS-389K variant had sufficient brightness to be characterized at the purified protein level (Supplementary Figure S14). Its relative molecular brightness was close to that of the parental GCaMP6s-BrUS, although the quantum yield was somewhat reduced, with a sufficiently increased extinction coefficient in the Sat form (Table 2); this variant also retained some fluorescence sensitivity to calcium, showing approximately an 11-fold increase in emission amplitude in response to an increase in free calcium concentration in the solvent from 0 to 39 µM (0–10 mM [CaEGTA]) (Figure 5A). However, the sensitivity of GCaMP6s-BrUS-389K in the nanomolar range was significantly reduced compared to the parental GCaMP6s-BrUS (signals at 0–7 mM [CaEGTA], or 0–350 nM free calcium were indistinguishable), and its fluorescence lifetime was insensitive to calcium (Figure 5B, Supplementary Figure S15).

To find new indicator variants with an increased molecular/cellular brightness and enhanced sensitivity to calcium, we conducted random mutagenesis on the template of all six aforementioned point mutants (M314G, D317S, D317G, R389G, R389T, and R389K). The primary screening of the resulting random libraries was based on selection by apparent fluorescence brightness (shown 24 h post transformation by bacterial colonies). For libraries generated by random mutagenesis, we screened approximately 100,000 colonies (10 standard Petri dishes) per round for each template coding sequence. After the first round of mutagenesis, no colonies meeting our primary requirements were found. Therefore, we conducted a second round of mutagenesis using the DNA obtained in the first round as a template. After the second round of random mutagenesis, five bright variants derived from the R389K-based library were revealed. These variants (see Supplementary Table S2) carried between 1 to 6 random amino acid substitutions per gene, and the overall mutagenesis efficiency was found to be 1–7 substitutions per 1000 nucleotides. Random mutagenesis of all other structure-guided point mutants did not yield colonies with brightness sufficient for further analysis. The selected clones (Supplementary Table S2) were expressed in a bacterial system, isolated, and subjected to the fluorescence measurement of their sensitivity to the calcium concentration in both the spectral and time domains.

**Figure 6:**
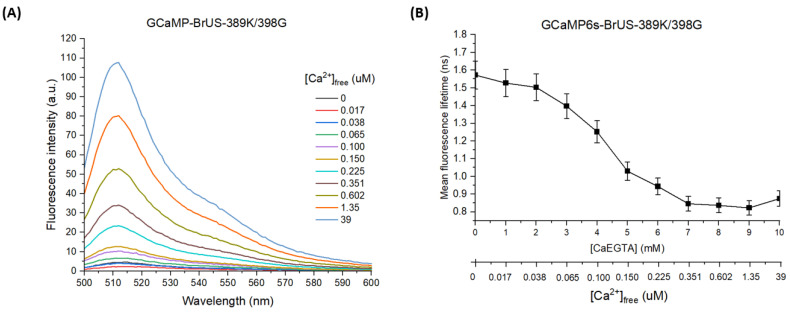
Calcium sensitivity of GCaMP6s-BrUS-389K/398G fluorescence. (**A**) The dependence of fluorescence intensity at 510 nm (λ_ex_ = 475 nm) on calcium concentration, expressed as [Ca^2+^]_free_. (**B**) The graph describing the dependence of the amplitude-weighted mean fluorescence lifetime on calcium concentration ([CaEGTA] and [Ca^2+^]_free_). λ_ex_ = 450 nm, repetition rate is 20 MHz. Standard errors of the mean (S.E.M.) are shown for each data point (*n* = 3).

Among the variants of GCaMP6s-BrUS selected after two rounds of mutagenesis, the only one that simultaneously met the criteria of satisfactory colony brightness 24 h after transformation, molecular brightness, and calcium sensitivity of fluorescence in both the spectral and time domain was GCaMP6s-BrUS-R389K/E398G (see Table 2 and Figure 6, Figures S16 and S17). According to our structural data on GCaMP6s-BrUS, the side chain of E398 is oriented at the interface between the beta-barrel and calmodulin domain, so its substitution might influence the dynamics of the interaction between these domains. With steady-state spectral properties almost identical to GCaMP6s (Supplementary Figure S7), it sufficiently (Table 2) surpassed the parental GCaMP6s-BrUS-389K in molecular brightness and the dynamic range of the calcium response (~27-fold vs. 11-fold change in fluorescence intensity, Figure 4A and Figure 5A). GCaMP6s-BrUS-389K/398G’s profile of fluorescence sensitivity to calcium was also found to be enhanced compared to its predecessor. Specifically, its calcium-dependent signal was clearly distinguishable among all analyte concentrations used (except 0 to 2 mM [CaEGTA]; Figure 6A). One can note the surprisingly even character of the signal change within the 5–10 mM [CaEGTA] range, which probably reflects the dissociation constant shift caused by the 398G substitution. More importantly, GCaMP-BrUS-389K/398G exhibited a well-pronounced mean fluorescence lifetime dependence on calcium concentration (Figure 6B), with approximately a 2-fold change (from 1.6 to 0.9 ns) in τ_m_ value upon a [CaEGTA] increase from 0 to 7 mM. Although, nominally, this lifetime contrast is inferior to that of GCaMP6s-BrUS-145 (see Figure 3 and Figure 5B), the ten-fold advantage in molecular brightness is likely to make its practical application for determining the absolute calcium concentration more effective by reducing the contribution (and corresponding systematic error) of cell autofluorescence and/or solvent fluorescence. Importantly, the fluorescence lifetime of GCaMP-BrUS-389K/398G was found to be insensitive to pH over a wide (and biochemically relevant) range of 5.5–8.5 (Supplementary Figure S18). Thus, this indicator variant is potentially capable of detecting the calcium cation in most biological model systems without bias caused by pH changes. The magnesium cation at a physiological concentration (20 mM), according to our measurements (Supplementary Figure S19), also did not affect the response of this indicator variant in the time domain.

The measurement of the absolute calcium concentration using time-resolved fluorescence microscopy remains a technique applicable only in specific experimental contexts. Imaging modalities that allow such measurements are slow; even in advanced time-resolved microscopes, the frame rate does not typically exceed 5 fps [49,50], and most commercial FLIM systems take between several seconds to a minute to scan the entire field of view [51,52,53]. Therefore, indicators such as GCaMP6s-BrUSLEE-389K/398G, Tq-Ca-FLITS [38], and, especially, GCaMP6s-BrUS-145 are barely suitable for monitoring calcium dynamics. It can be assumed that these indicators will find application as a tool complementing fast intensity-based measurements in neurobiological research, allowing for the collection of a larger density of information per sample without the need for additional spectral channels.

GCaMP6s-BrUS may find application in high-speed volumetric imaging, providing a potential two- to three-fold increase in the data acquisition rate compared to conventional GCaMP family indicators. This variant is the first genetically encoded calcium indicator with a sub-nanosecond lifetime, and it possesses some shortcomings originating from its molecular design. In particular, it retains some lifetime sensitivity to calcium, which may affect the quality of intensity-based measurements in light beads microscopy [42]. Additionally, compared to GCaMP6s (and especially to newer CaMPs [5,18,54]), CaMP6s-BrUS has a reduced sensitivity to calcium and a dynamic range of response, which can considerably reduce the sensing efficiency in high-speed data acquisition in thick samples. Finally, its molecular brightness is expectedly modest, which, however, is unlikely to be improved much given its short fluorescence lifetime.

Given the great demand for calcium indicators in neuroscience, where, in recent years, the preferred approach has become in vivo calcium imaging [5,8,55], including in the nervous tissue of living mammals, the properties of the label in the two-photon excitation mode come to the forefront [56,57,58,59]. Since, for many fluorophores, the profiles of one-photon and two-photon absorption differ markedly [60], the specifications of the indicator measured in the one-photon mode cannot be extrapolated to the two-photon mode. Our study does not cover the two-photon properties of the engineered indicators, so the potential for their application in the depth of nervous tissue in combination with multiphoton microscopy modalities remains unclear.

Nominally, GCaMP6s-BrUS-145 has the widest dynamic range (DR) of fluorescent response among lifetime-based indicators (1.5–1.6 ns). However, we assume that the brighter Tq-Ca-FLITS (DR of 1.3 ns) and GCaMP6s-BrUSLEE-389K/398G (DR of 0.7 ns) will have an advantage in cellular imaging due to the lower influence of autofluorescence on their signal and the faster collecting of a sufficient number of photons for quantitative analysis. An important advantage of the new indicators over their only direct competitor, the cyan-emitting Tq-Ca-FLITS, is the potentially lower cytotoxicity of the excitation light source. Additionally, the fluorescence lifetime of GCaMP6s-BrUSLEE-389K/398G is somewhat less dependent on pH in mildly acidic conditions, and its dynamic range in the intensity-based mode is almost an order of magnitude wider than that of Tq-Ca-FLITS, which opens up the prospect of its ‘dual-mode’ application (calcium dynamics + measurement of absolute concentration in stationary conditions).

## 3. Materials and Methods

### 3.1. Genetic Engineering

#### 3.1.1. Genetic Engineering of GCaMP6s-BrUS and GCaMP6s-BrUS-145 Constructs

The GCaMP6s-BrUS and GCaMP-BrUS coding sequences were generated using the overlap-extension PCR technique.

For the 1st step of the overlap-extension PCR method, we amplified two gene parts separately in two tubes: from the 5′-end to the putative site of mutation and from the site of mutation to the 3′-end. For amplification, primers containing the desired mutation were used (Table 3).

In addition, the primers used to amplify different gene parts contained complementary parts. For the 2nd step, the amplified gene parts were mixed, hybridized at the annealing temperature of the complementary sequences, and “completed” to a full-length gene with the desired mutation using primerless PCR for several cycles. Without visualizing using electrophoresis, the full-length product was additionally amplified by adding the terminal primers to the reaction (the 3rd step of the overlap-extension PCR) and cloned into the pQE-30 vector (Qiagen, Germantown, MD, USA) at the BamHI and HindIII sites.

The PCR program for the amplification of the gene parts was as follows: 95 °C for 5 min, followed by 18 cycles of (95 °C for 30 s, 54 °C for 30 s, and 72 °C for 60 s), and a final extension at 72 °C for 7 min. The overlap PCR program was as follows: 95 °C for 5 min, followed by 20 cycles of (95 °C for 30 s, 61 °C for 30 s, and 72 °C for 30 s), and a final extension at 72 °C for 7 min. The PCR program for the 3rd step was as follows: 95 °C for 5 min, followed by 20 cycles of (95 °C for 30 s, 55 °C for 30 s, and 72 °C for 2 min), and a final extension at 72 °C for 7 min.

#### 3.1.2. Site-Directed Mutagenesis of GCaMP6s-BrUS

The GCaMP6s-BrUS point mutants were generated using the IVA PCR technique [61] with the following oligonucleotide primers containing the appropriate substitution (Table 4).

The PCR reaction was conducted using Phusion^®^ High-Fidelity DNA polymerase (NEB, Ipswich, MA, USA), according to the following protocol: 30 s at 95 °C, 18 cycles of (10 s at 95 °C, 30 s at 58 °C, and 4 min at 72 °C), and a final 7 min extension at 72 °C. The addition of 1 μL FastDigest DpnI enzyme (Thermo Fisher Scientific, Waltham, MA USA) was followed by a 20 min incubation at 37 °C prior to transformation. The obtained mutants were cloned into the Y514 vector backbone (Cloning facility, Moscow, Russia).

#### 3.1.3. Random Mutagenesis by PCR

For error-prone PCR, 0.1 ng of template was used along with 10× Taq Turbo buffer, with final Mg^2+^ concentration of 2.5 mM (Evrogen, Moscow, Russia), dCTP and dTTP at 1 mM (final concentration), dATP and dGTP at 0.2 mM (final concentration), primers (forward 5′-ATCGGAATTCATTAAAGAGGAGAAATTAACTATGGGC-3′/reverse 5′-ATCGACCGGTGGCGTACATTTTCAAAGAGTGC-3′) at 10 µM (final concentration), and Taq DNA polymerase (Evrogen, Moscow, Russia).

The PCR program for amplification was as follows: 95 °C for 5 min, followed by 18 cycles of (95 °C for 30 s, 59 °C for 30 s, and 72 °C for 2.5 min), and a final extension at 72 °C for 7 min.

The amplified product was cloned into the Y514 vector (Cloning facility, Moscow, Russia) at the EcoRI and BshTI sites. *E. coli* XL1-Blue (Evrogen, Moscow, Russia) cells were transformed with the Y514 plasmids containing the libraries and grown overnight.

#### 3.1.4. Screening of Random Variant Libraries

The primary step of screening was carried out by assessing the fluorescence intensity of the obtained random mutants. The fluorescence of bacterial colonies was analyzed 24 h post-transformation under an Olympus SZX12 fluorescence binocular (Olympus, Tokyo, Japan), and the clonal variants showing fluorescence intensity higher than the parental variant were selected for subsequent analysis. To this end, lysogeny broth (LB) cell growth medium (100 mL in 750 mL conical flasks) was supplemented with ampicillin (200 µg/mL) and inoculated with a selected single colony from a Petri dish 24 h after cell transformation. Cell biomasses were incubated at 37 °C for 16 h at 230 rpm, followed by 25 °C for 24 h at 180 rpm for protein maturation. Proteins were isolated, and the sensitivity of their fluorescence intensity and lifetime to calcium concentration was assessed using a set of buffers from the Calcium Calibration Buffer Kit #1 (C3008MP, Invitrogen, Waltham, MA USA).

### 3.2. Protein Expression, Purification, and Crystallization

#### 3.2.1. Protein Expression and Isolation

GCaMP6s, GCaMP6s-BrUS, and GCaMP6s-BrUS-145 coding sequences in the pQE-30 (Qiagen, Venlo, The Netherlands) vector backbone, and the coding sequences of the remaining variants in the Y514 vector backbone (Cloning facility, Moscow, Russia) were expressed in the *E. coli* XL1-Blue strain (Evrogen, Moscow, Russia). The expression was run at 37 °C for 16 h at 230 rpm and then at 25 °C for 24 h at 180 rpm for protein maturation. After centrifugation, the cell pellets were resuspended in PBS (pH 7.4, Gibco, ThermoFisher Scientific, Waltham, MA, USA) and kept in an ice-water bath. The bacterial biomass was treated with ultrasound for 15 min total treatment time (45 cycles of 5 s sonication at 30% input power amplitude and 15 s in off-cycle) using a Sonics Dismembrator (Fisher Scientific, Pittsburgh, PA, USA). TALON metal-affinity resin (Clontech Laboratories, Takara Bio USA, Inc., Palo Alto, CA, USA) was used to isolate the proteins.

#### 3.2.2. Protein Purification

##### Immobilized Metal Affinity Chromatography

A 15 mL glass column containing 1 mL of TALON resin was washed with 10 mL of PBS (10× volume) to pre-equilibrate the column. The bacterial lysate supernatant obtained after centrifugation at 12,000× *g* for 15 min at 4 °C was incrementally loaded onto the column. The resin with bound proteins was rinsed with 10× volume of PBS and washed with 10× volume of 10 mM imidazole in PBS. Final elution was performed with 5× volume of 250 mM imidazole in PBS, collecting 0.5 mL fractions.

Fractions containing eluted proteins were pooled based on their absorbance at 280 nm, measured using a Cary 100 UV–Visible spectrophotometer (Agilent, Santa Clara, CA, USA). Desalting was carried out by ultrafiltration using an Amicon Ultra, 10 kDa, 0.5 mL filter unit (Ultracel) against a PBS buffer, resulting in a final imidazole concentration far below 1 mM.

##### Anion-Exchange Chromatography

GCaMP6s-BrUS and GCaMP6s-BrUS-145 protein samples after immobilized metal affinity chromatography were applied to a Q Sepharose HiTrap Fast Flow 1 mL column (GE Healthcare, Danderyd, Sweden), equilibrated and washed with 50 mM Tris-HCl buffer, with pH 8.8 at a flow rate of 1 mL/min. The elution was carried out by linear NaCl gradient from 0 to 1 M (10 mL), and 1 mL fractions were collected. Buffer gradient formation and automatic fraction collection was controlled with a Shimadzu SCL10AVP chromatography system (Shimadzu, Kyoto, Japan) equipped with a SPD10AVP diode array detector. After elution, fractions containing protein of interest were combined and concentrated using a 50 kDa Amicon^®^ Ultra centrifugal filter unit (Merck Millipore, Darmstadt, Germany). The concentrated samples were used for size-exclusion chromatography.

##### Size-Exclusion Chromatography

GCaMP6s-BrUS and GCaMP6s-BrUS-145 containing samples collected and concentrated after anion exchange chromatography were applied to a Superdex 200 10/300 GL column (GE Healthcare, Sweden) on a Shimadzu SCL10AVP chromatography system (Shimadzu, Japan). The column was operated at room temperature at a flow rate of 0.4 mL/min with a 50 mM sodium phosphate buffer, pH 7.4. During elution, 0.5 mL fractions were collected. Fractions, containing protein of interest, were combined and concentrated on a 50 kDa Amicon^®^ Ultra centrifugal filter unit (Merck Millipore, Germany). The concentrated samples of GCaMP6s-BrUS and GCaMP6s-BrUS-145 were maintained at +4 °C and aliquots were used in the subsequent gel electrophoresis experiments.

##### Denaturing Polyacrylamide Gel Electrophoresis

SDS-PAGE of the samples was performed using a 4–20% Mini-PROTEAN^®^ TGX Stain-Free™ Protein Gels (#4568094, Bio-Rad, Hercules, CA, USA), in accordance with the manufacturer’s recommendations. PageRuler™ Unstained Broad Range Protein Ladder (Thermo Scientific, Waltham, MA, USA) was used as a protein molecular weight marker. The visualization of protein bands was performed using the stain-free protocol on the ChemiDoc MP Imaging System (Bio-Rad, USA).

#### 3.2.3. Protein Crystallization

A 96-well format crystallization screening of GCaMP6s-BrUS and GCaMP6s-BrUS-145 was performed with a robotic crystallization system Oryx4 (Douglas Instruments, Hungerford, Berkshire, UK) and commercially available crystallization screens (Hampton Research, Aliso Viejo, CA, USA) using sitting drop vapor diffusion method at 288 K. Proteins concentrated at 10 mg/mL in a buffer that contained 25 mM borate buffer, with pH 9.5 and 150 mM NaCl, were saturated with 39 μM calcium ions prior to crystallization. Protocol with a final drop volume of 0.3 μL of mixed volumes of protein and reservoir in the 1:2, 1:1, and 2:1 ratio was used. The yellow-colored crystals of GCaMP6s-BrUS and GCaMP6s-BrUS-145 were obtained in the following conditions: 0.2 M Ammonium tartrate dibasic pH 7.0; 20% PEG 3350 and 0.2 M Sodium thiocyanate pH 6.9; and 20% PEG 3350, respectively, and used for the X-ray experiment.

### 3.3. X-Ray Data Processing Structure Solution and Refinement

Crystals of GCaMP6s-BrUS and GCaMP6s-BrUS-145 were soaked in a reservoir solution containing 20% ethylene glycol as a cryoprotectant immediately before data collection. The diffraction data for GCaMP6s-BrUS were collected on the Manaca beamline at LNLS (Campinas, Brazil) at 100 K, while data for GCaMP6s-BrUS-145 was collected using XtaLAB Synergy-S (Rigaku, Tokyo, Japan) at the same temperature. Crystals of both proteins belonged to the space group P4_1_2_1_2 with one protein molecule per asymmetric unit. The data were processed with XDS [62] and CrysAlisPro v41.93a (Supplementary Table S3).

The structure of GCaMP6s-BrUSLEE was solved via molecular replacement method using MOLREP v. 11.9.02 [63] and the GCaMP2 (PDB ID—3EK4) structure as a starting model. The structure of GCaMP6s-BrUSLEE-145 was solved with the same technique using GCaMP6s-BrUSLEE as a model. Data processing was performed using the CCP4 suite [64]. The refinement of the model was carried out with REFMAC5 v.5.8.0430 [65]. TLS parametrization, covalent and metal link restraints, and isotropic B-factors approximation were used during the refinement process. The manual correction of the models was carried out with COOT [66]. The refinement statistics are summarized in Supplementary Table S3.

In the structure of GCaMP6s-BrUS, the polypeptide chain had no electron density commencing before A45 residue and after T461 residue. Twelve amino acids (residues 158–169) including the mobile linker GGTGGS joining the ex termini of the permutant were not distinguishable in the electron density. The distal linker joining the EGFP and CaM domains comprising the ^314^MLPDQL^319^ sequence was also poorly distinguishable on the electron density map. Similarly, the polypeptide chain of GCaMP6s-BrUS-145 had no electron density before M47 and after T460 residues. Fourteen amino acids (residues 156–169) joining the ex termini of the permutant were not distinguishable in the electron density map. The distal linker joining the EGFP and CaM domains comprising the LPDQL (with the methionine residue deletion) sequence had a poor electron density map.

### 3.4. Absorption and Fluorescence Spectroscopy

#### 3.4.1. General Information and Full-Spectra Recording

Using a Cary100 UV/VIS spectrophotometer and a Cary Eclipse fluorescence spectrophotometer (Agilent Technologies, Santa Clara, CA, USA), the absorbance and fluorescence spectra of proteins were measured. Phosphate-buffered saline (PBS, pH 7.4, Gibco), zero free calcium buffer, and 39 μM free calcium buffer (C3008MP, Invitrogen, USA) were used to dilute the samples with equal protein concentrations. The measurement range for their absorption spectra was 350–600 nm. Fluorescence excitation spectra were recorded at λ_em_ = 525 nm, and fluorescence emission spectra were recorded at λ_ex_ = 475 nm. The absorbance of the proteins was set to be less than 0.1 for the fluorescence recordings.

To determine the fluorescence quantum yield, the fluorescence of the proteins under study was directly compared to that of the reference fluorophore, EGFP fluorescent protein with a known absolute fluorescence quantum yield of 0.6 [67], and corrected to the area under the emission spectrum according to the following formula:

$${FQY}_{s}={F{QY}_{EGFP}\times \frac{S_{s}}{S}}_{EGFP}$$

Subscripts “s” and “EGFP” indicate the sample and the reference EGFP, respectively.

The spectral area (S) under emission spectra was calculated by integration between 450 and 650 nm. For the fluorescence quantum yield measurements, aqueous solutions of the sample, and reference with the same absorbance (typically, 0.05) at an absorption maximum of anionic GFP-type chromophore (490–508 nm) were used. The sample and reference emission spectra were recorded using the same instrument settings (ex/em monochromator slits, PMT voltage, dwelling time, etc.).

Based on measurements of the absorbance spectra of the proteins in their native and alkali-denatured states (450 nm peak with an extinction coefficient of 44,000 M^−1^cm^−1^), extinction coefficients for proteins were calculated.

#### 3.4.2. Time-Resolved Fluorescence Spectroscopy

A time-resolved miniTau fluorescence spectrometer (Edinburgh Instruments, Livingston, UK) was used to perform measurements. Data were captured within a 50 ns window divided into 2048 time channels. An EPL-450 picosecond laser (Edinburgh Instruments), featuring a central emission wavelength of 445.6 nm and a repetition rate of 20 MHz, provided fluorescence excitation. Photon counting occurred within the 475–525 nm spectral range. Fluoracle 2.5.1 software (Edinburgh Instruments) was utilized for data processing, visualization, and Pearson’s test determination (χ^2^).

### 3.5. Calcium Sensitivity Measurements

To determine the calcium sensitivity of proteins, a set of buffers from the Calcium Calibration Buffer Kit #1 (C3008MP, Invitrogen, USA) was used. For sample preparation, aliquots of proteins were dissolved in the buffer at a 1:200 *v*/*v* ratio to a final concentration of 5 μg/mL. The signals of the probes were measured as the intensity of the fluorescence of the emission peak at λ_em_ = 525 nm (λ_ex_ = 475 nm) or as a fluorescence lifetime measured using a time-resolved miniTau fluorescence spectrometer (Edinburgh Instruments, Livingston, UK).

To determine the impact of magnesium cations on the calcium sensitivity of GCaMP-BrUS-389K/398G, MgCl_2_ was added to a set of buffers from the Calcium Calibration Buffer Kit #1 (C3008MP, Invitrogen, USA) to a final concentration of 20 mM. For sample preparation, aliquots of proteins were dissolved in the buffer at a 1:200 *v*/*v* ratio to a final concentration of 5 μg/mL. The signals of the probes were measured as a fluorescence lifetime measured using a time-resolved miniTau fluorescence spectrometer (Edinburgh Instruments, Livingston, UK).

## 4. Conclusions

In this study, we describe new variants of calcium indicators based on the well-established GCaMP6 backbone and the green fluorescent protein BrUSLEE. One of them, named GCaMP6s-BrUS, like the ancestral protein BrUSLEE, demonstrates a sub-nanosecond fluorescence lifetime while maintaining an acceptable molecular brightness. Its calcium sensitivity in the intensiometric mode and the small calcium-dependent changes in its fluorescence lifetime make it a candidate for use in light beads microscopy and other high-throughput microscopy modalities where the data collection speed is limited by the fluorescence decay kinetics. The practical value of GCaMP6s-BrUS is somewhat reduced by the slight residual sensitivity of its fluorescence lifetime to calcium and its moderate dynamic range of response. These properties are subject to improvement in future work.

The other two variants, on the contrary, exhibit dynamic, calcium-sensitive behavior in the time domain. For example, GCaMP6s-BrUS-145 has the largest fluorescence lifetime contrast between the so-called Apo and Sat forms of the indicator described so far (both in relative and absolute terms). However, its extremely low molecular brightness is likely to hinder its successful application in biological model systems. The variant GCaMP-BrUS-389K/398G, obtained through semi-rational engineering based on a comparative analysis of the spatial structures of GCaMP6s-BrUS and GCaMP6s-BrUS-145, also has a pronounced calcium sensitivity of its fluorescence lifetime. However, it is significantly brighter than GCaMP6s-BrUS-145 and has a better spectral sensitivity to the analyte. A noticeable drawback of both GCaMP6s-BrUS-145 and GCaMP6s-BrUSLEE-389K/398G is the inverse proportional dependence of their lifetime on the calcium concentration.

The structural data obtained in our study may serve as a starting point for engineering new, improved GECI variants with a calcium-sensitive fluorescence lifetime. Moreover, the GCaMP6s-BrUS family indicators themselves are subject to further optimization to increase the molecular brightness and/or the range of fluorescence lifetime changes. A possible strategy for advancing in these directions could be saturation mutagenesis at already identified key amino acid positions followed by optimization through random mutagenesis. Additionally, further experiments are needed to determine the effectiveness of these indicators in multiphoton microscopy.

## Figures and Tables

**Table 1:** Fluorescent properties of GCaMP6s, GCaMP6s-BrUS, and GCaMP6s-BrUS-145 proteins. The data for each protein were measured in both their calcium-free (Apo) and calcium-saturated (Sat) forms.

Protein	Form	λ_ex_/λ_em_, nm	FQY *	EC ** (M^−1^cm^−1^)	RB ***, %
GCaMP6s	Apo	(400 and 508)/515	0.42 ± 0.01	8220 ± 411	10.2 ± 0.6
	Sat	497/509	0.56 ± 0.03	76,557 ± 827	130 ± 7
GCaMP6s-BrUS	Apo	(400 and 501)/515	0.124 ± 0.006	17,479 ± 735	5.3 ± 0.4
	Sat	492/510	0.21 ± 0.01	70,884 ± 854	45 ± 6
GCaMP6s-BrUS-145	Apo	(400 and 500)/515	0.023 ± 0.001	4676 ± 234	0.28 ± 0.01
	Sat	493/512	0.131 ± 0.006	44,850 ± 442	13.6 ± 3.2

* FQY—fluorescence quantum yield; ** EC—extinction coefficient (absorption peak at 500 nm); *** RB—relative brightness (molecular brightness compared to that of EGFP, having absolute FQY of 0.6 and EC = 55,000 M^−1^cm^−1^).

**Table 2:** Fluorescent properties of GCaMP6s-BrUS-389K and GCaMP6s-BrUS-389K/398G proteins. The data for each protein were measured in both their calcium-free (Apo) and calcium-saturated (Sat) forms.

Protein	Form	λ_ex_/λ_em_	FQY *	EC **	RB ***, %
GCaMP6s-BrUSLEE-389K	Apo	(392 and 501)/511	0.076 ± 0.002	6190 ± 115	1.12 ± 0.02
	Sat	494/510	0.12 ± 0.01	83,463 ± 417	28 ± 2
GCaMP6s-BrUSLEE-389K/398G	Apo	(398 and 502)/515	0.22 ± 0.01	7373 ± 168	5.2 ± 0.4
	Sat	496/512	0.30 ± 0.01	123,509 ± 354	112 ± 5

* FQY—fluorescence quantum yield; ** EC—extinction coefficient; *** RB—relative brightness (molecular brightness compared to that of EGFP, having absolute FQY of 0.6 and EC = 55,000 M^−1^cm^−1^).

**Table 3:** Oligonucleotides used for the overlap-extension PCR.

Substitution	Primers for the 1st Step of the Overlap-Extension PCR	Primers for the 3rd Step of the Overlap-Extension PCR
T65G	forward 5′-ATGCGGATCCATGGCTAGCATGACTGGTGGACA-3′/reverse 5′-CACGCCGTAGCCCAGGGTGGT-3′	forward 5′-ATGCGGATCCATGGCTAGCATGACTGGTGGACA-3′/reverse 5′-ATGCAAGCTTTCACTATTACTTCGCTGTCATCATTTGTACAAAC-3′
	forward 5′-GGCTACGGCGTGCAGTGCTTCAGC-3′/reverse 5′-ATGCAAGCTTTCACTATTACTTCGCTGTCATCATTTGTACAAAC-3′	
Y145M	forward 5′-ATGCGGATCCATGGCTAGCATGACTGGTGGACA-3′/reverse 5′-CAGTTGGTCCGGCATGTTGTACTCCAGCTT-3′	
	forward 5′-CTGGAGTACAACATGCTGCCGGACCAACTG-3′/reverse 5′-ATGCAAGCTTTCACTATTACTTCGCTGTCATCATTTGTACAAAC-3′	
F165Y	forward 5′-ATGCGGATCCATGGCTAGCATGACTGGTGGACA-3′/reverse 5′-GCGGATGTGGTAGTTCGCCTT-3′	
	forward 5′-TACCACATCCGCCACAACATCGAG-3′/reverse 5′-ATGCAAGCTTTCACTATTACTTCGCTGTCATCATTTGTACAAAC-3′	
deletion Y145	forward 5′-ATGCGGATCCATGGCTAGCATGACTGGTGGACA-3′/reverse 5′-CAGTTGGTCCGGGTTGTACTCCAGCTT-3′	
	forward 5′-AAGCTGGAGTACAACCTGCCGGACCAACTG-3′/reverse 5′-ATGCAAGCTTTCACTATTACTTCGCTGTCATCATTTGTACAAAC-3′	

**Table 4:** Oligonucleotides used for the IVA mutagenesis.

Substitution	Forward Primer	Reverse Primer
M314G	5′-ACAAGCTGGAGTACAACGGCCTGCCGGACCAACTGACTGAAG-3′	5′-GTTGTACTCCAGCTTGTGCCCCAGGATGTTGCCGTCC-3′
D317S	5′-GTACAACATGCTGCCGAGCCAACTGACTGAAGAGCAGATCGCAG-3′	5′-CGGCAGCATGTTGTACTCCAGCTTGTGCCCCAGGATGTT-3′
D317G	5′-GTACAACATGCTGCCGGGCCAACTGACTGAAGAGCAGATCGCAG-3′	5′-CGGCAGCATGTTGTACTCCAGCTTGTGCCCCAGGATGTT-3′
R389G	5′-TCCTGACAATGATGGCAGGCAAAATGAAATACAGGGACACGGAAGAAGAAATTAGAG-3′	5′-TGCCATCATTGTCAGGAACTCAGGGAAGTCGATTGTGCC-3′
R389K	5′-TCCTGACAATGATGGCAAAAAAAATGAAATACAGGGACACGGAAGAAGAAATTAGAG-3′	5′-TGCCATCATTGTCAGGAACTCAGGGAAGTCGATTGTGCC-3′
R389T	5′-TCCTGACAATGATGGCAACCAAAATGAAATACAGGGACACGGAAGAAGAAATTAGAG-3′	5′-TGCCATCATTGTCAGGAACTCAGGGAAGTCGATTGTGCC-3′

## Data Availability

The data presented in this study are available within the manuscript and Supplementary Materials. The raw data are available upon request from the corresponding authors.

## Supplementary Materials

The following supporting information can be downloaded at: https://www.mdpi.com/article/10.3390/ijms252312493/s1.
