# Supplementary material for: Calcium Indicators with Fluorescence Lifetime-Based Signal Readout: A Structure–Function Study
Source: Int J Mol Sci. 2024 Nov 21;25(23):12493. doi: 10.3390/ijms252312493 (PMC11640911; doi:10.3390/ijms252312493)
Supplement: Supplementary file 1 [file ijms-25-12493-s001.zip › ijms-3307967-supplementary.pdf]

Supplementary Materials

Calcium Indicators with Fluorescence Lifetime-Based Signal Readout: A Structure-Function Study

Tatiana R. Simonyan [1,†], Larisa A. Varfolomeeva [2,†], Anastasia V. Mamontova [1], Alexey A. Kotlobay [1], Andrey Y. Gorokhovatsky [1], Alexey M. Bogdanov [1,3,*] and Konstantin M. Boyko [2,*]

[1] Shemyakin-Ovchinnikov Institute of Bioorganic Chemistry, 117997 Moscow, Russia
[2] A.N. Bach Institute of Biochemistry, Research Centre of Biotechnology of the Russian Academy of Sciences, Moscow 119071, Russia
[3] Department of Photonics, İzmir Institute of Technology, İzmir 35430, Turkey
* Correspondence: noobissat@ya.ru (A.M.B.); kmb@inbi.ras.ru (K.M.B.)
† These authors contributed equally to this work.

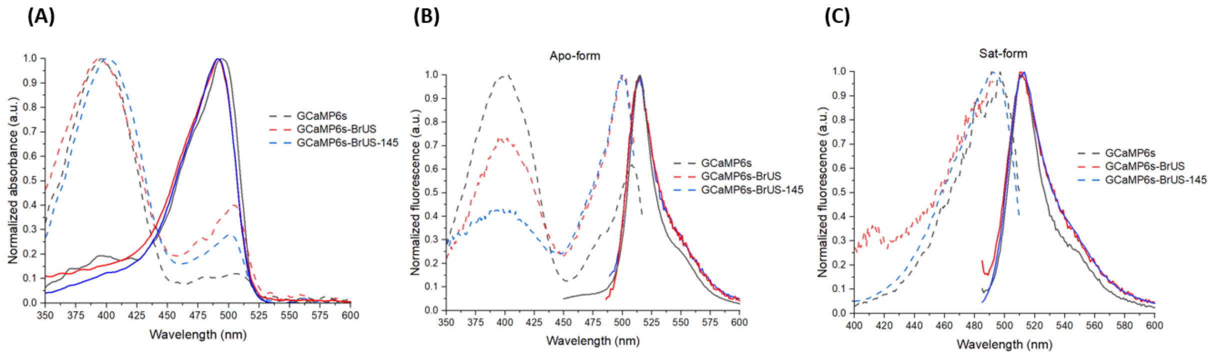

Supplementary Figure S1. Absorption **(A)** and fluorescence spectra of the purified GCaMP6s, GCaMP-BrUSLEE, and GCaMP-BrUSLEE-145 proteins in both their calcium-free (Apo) **(B)** and calcium-saturated (Sat) **(C)** forms. In the absorbance graph, dashed lines show absorbance spectra for the Apo form, solid lines – Sat form. In the fluorescence graphs, dashed lines show the excitation spectra (λ_{em} = 525 nm), solid lines – emission spectra (λ_{ex} = 400 nm for the Apo form, λ_{ex} = 475 nm for the Sat form).

Supplementary Table S1. Correspondence between [CaEGTA] and free calcium concentration.

[CaEGTA], mM	$[Ca^{2+}]_{free}$, µM
0	0
1	0.017
2	0.038
3	0.065
4	0.100
5	0.150
6	0.225
7	0.351
8	0.602
9	1.35
10	39

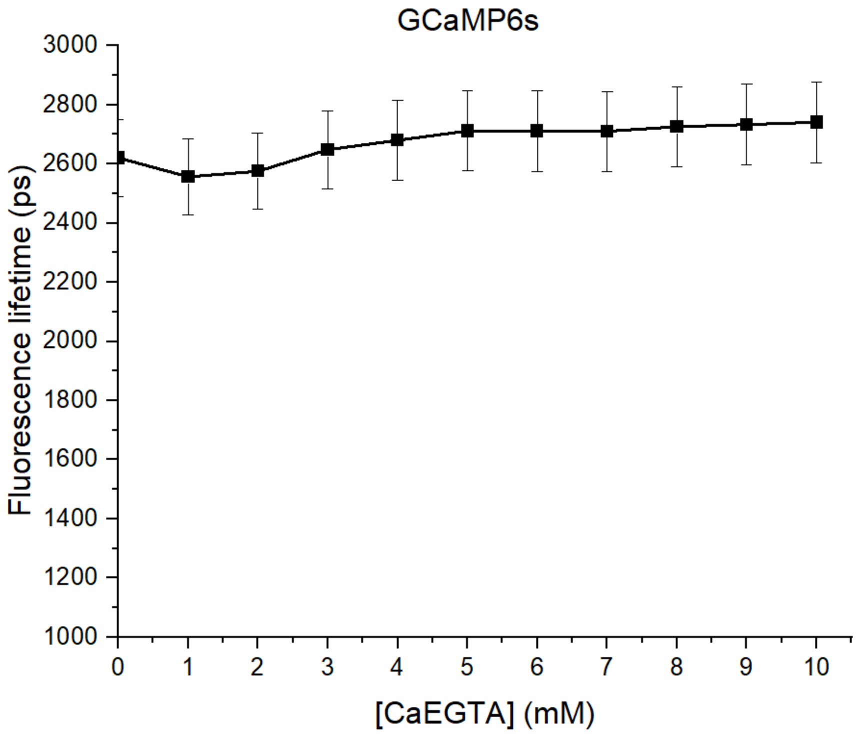

Supplementary Figure S2. Fluorescence decay kinetics of GCaMP6s fitted by a monoexponential model, recorded at various calcium concentrations upon excitation with a 450 nm picosecond laser.

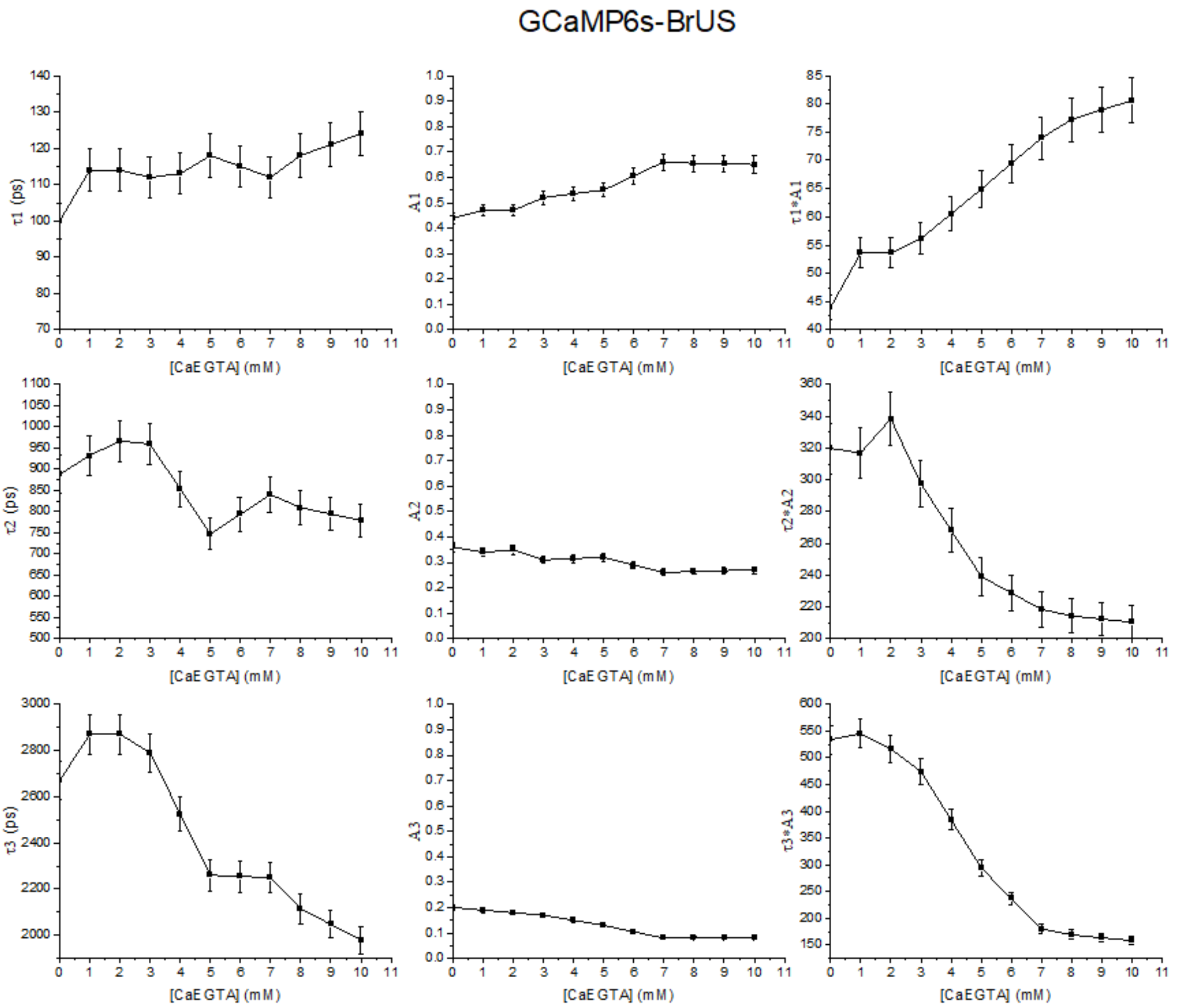

Supplementary Figure S3. Fluorescence decay kinetics of GCaMP6s-BrUS fitted by a triexponential model, recorded at various calcium concentrations upon excitation with a 450 nm picosecond laser.

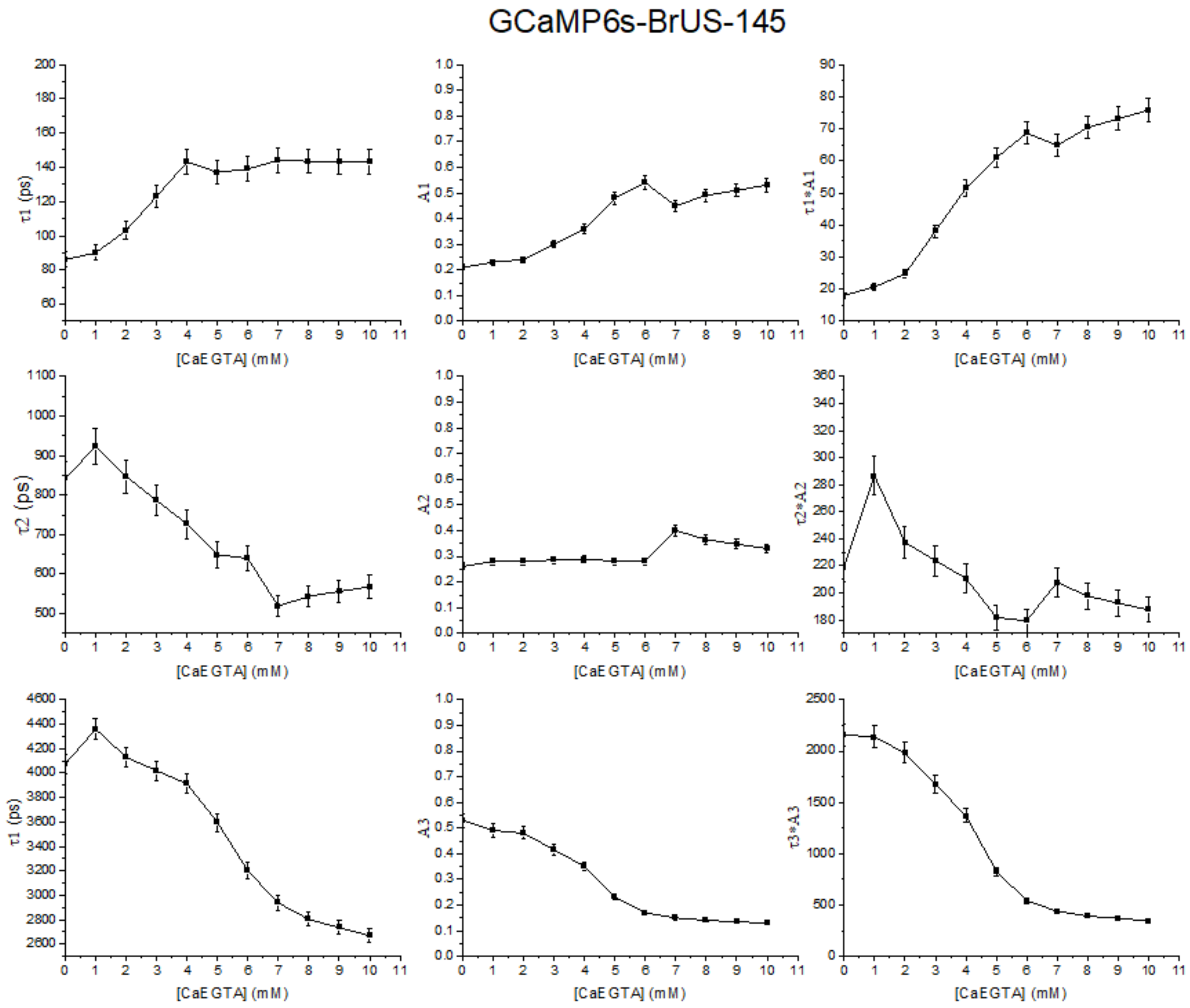

Supplementary Figure S4. Fluorescence decay kinetics of GCaMP6s-BrUS-145 fitted by a triexponential model, recorded at various calcium concentrations upon excitation with a 450 nm picosecond laser.

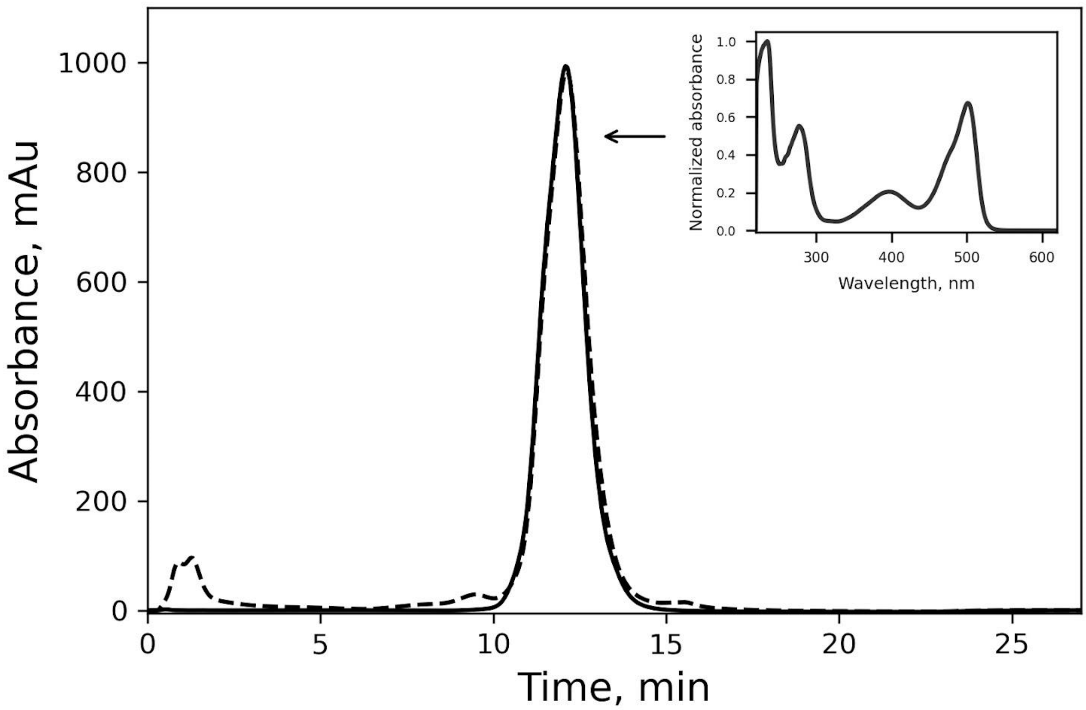

Supplementary Figure S5. Chromatographic profiles of GCaMP6s-BrUS batch 1 on Q Sepharose HiTrap Fast Flow 1 mL column monitored at wavelengths 280 nm (dashed line) and 488 nm (solid line). Inset: normalized absorption spectrum of GCaMP6s-BrUS.

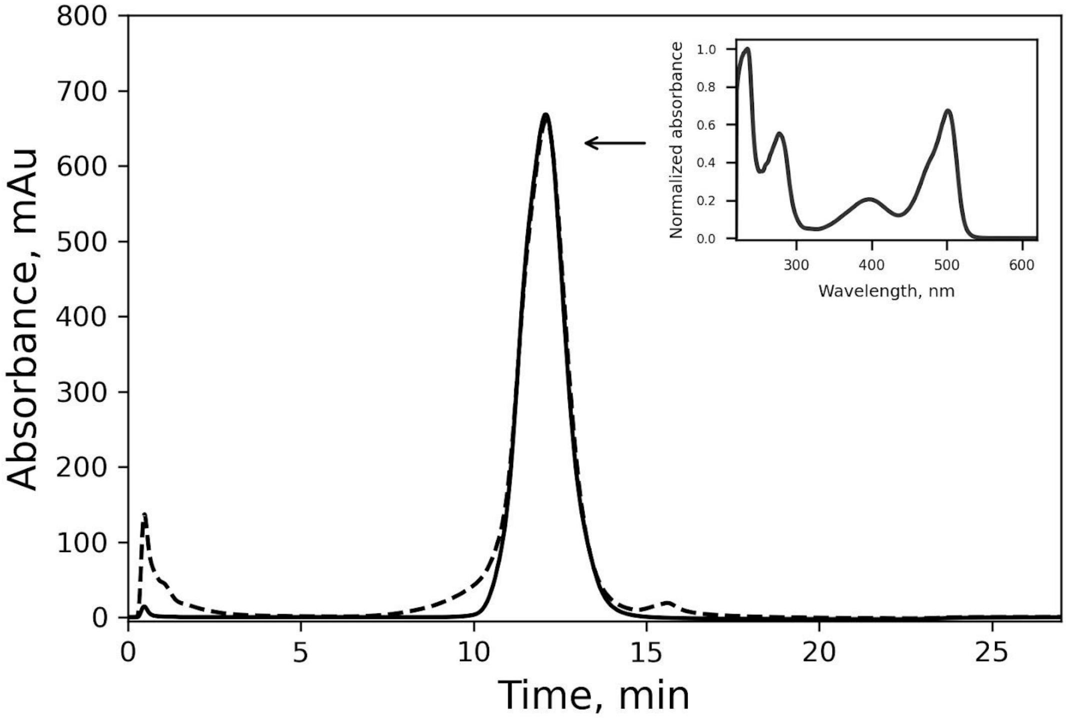

Supplementary Figure S6. Chromatographic profiles of GCaMP6s-BrUS batch 2 on Q Sepharose HiTrap Fast Flow 1 mL column monitored at wavelengths 280 nm (dashed line) and 488 nm (solid line). Inset: normalized absorption spectrum of GCaMP6s-BrUS.

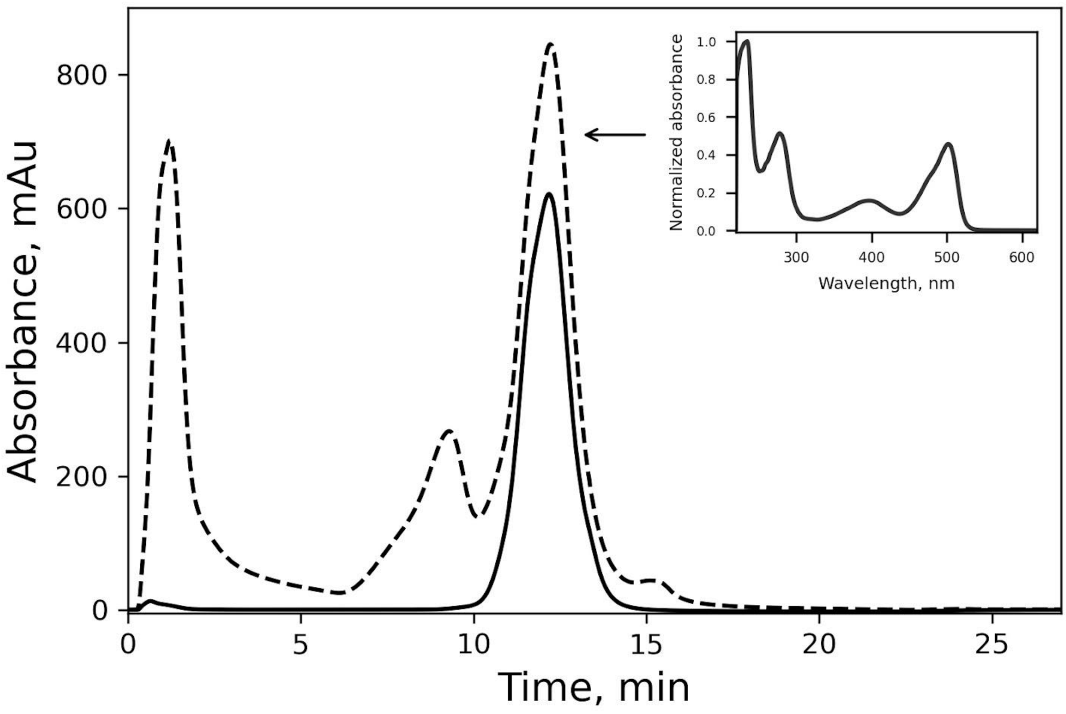

Supplementary Figure S7. Chromatographic profiles of GCaMP6s-BrUS-145 batch 1 on Q Sepharose HiTrap Fast Flow 1 mL column monitored at wavelengths 280 nm (dashed line) and 488 nm (solid line). Inset: normalized absorption spectrum of GCaMP6s-BrUS-145.

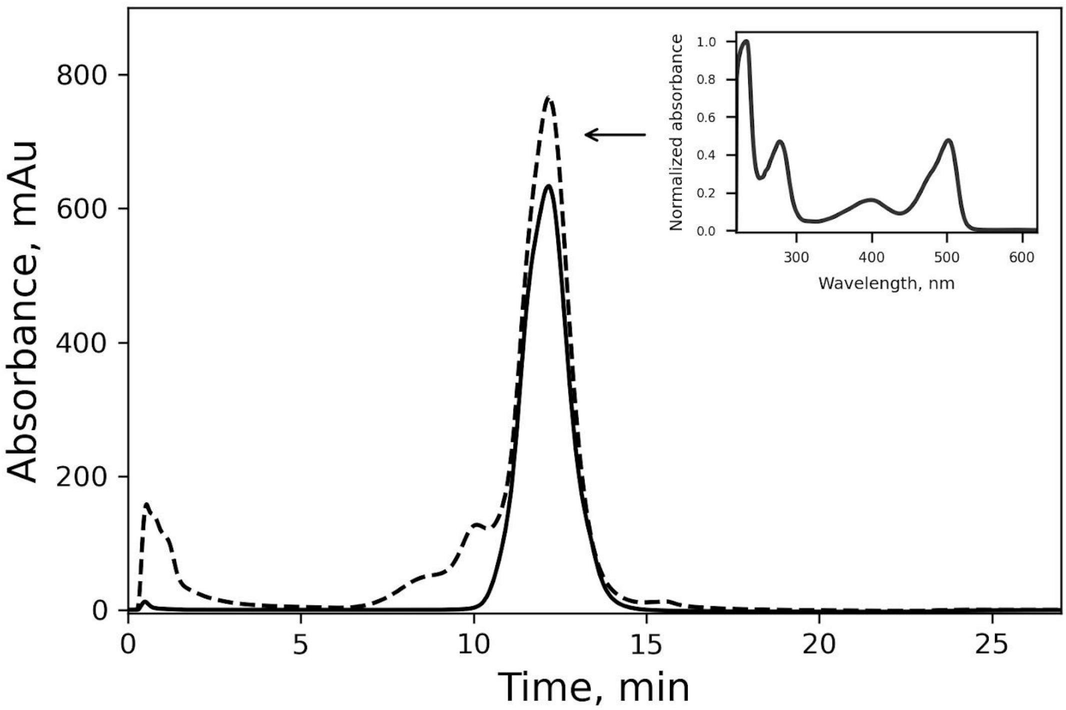

Supplementary Figure S8. Chromatographic profiles of GCaMP6s-BrUS-145 batch 2 on Q Sepharose HiTrap Fast Flow 1 mL column monitored at wavelengths 280 nm (dashed line) and 488 nm (solid line). Inset: normalized absorption spectrum of GCaMP6s-BrUS-145.

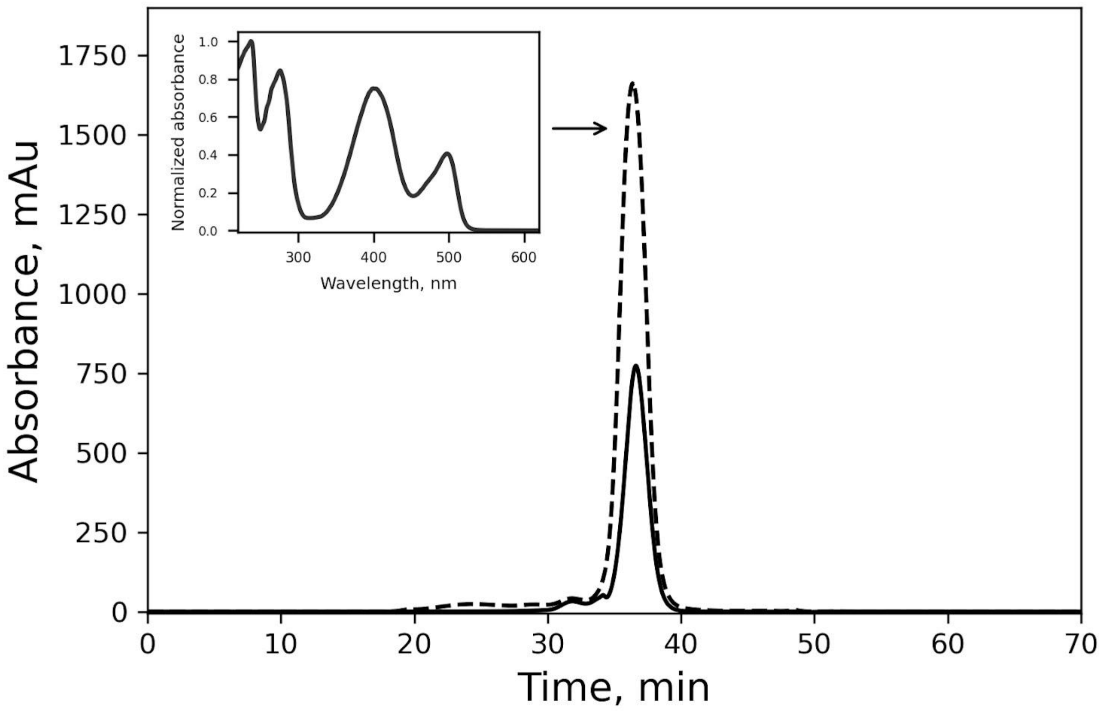

Supplementary Figure S9. Chromatographic profiles of GCaMP6s-BrUS batch 1 on Superdex 200 column monitored at wavelengths 280 nm (dashed line) and 488 nm (solid line). Inset: normalized absorption spectrum of GCaMP6s-BrUS.

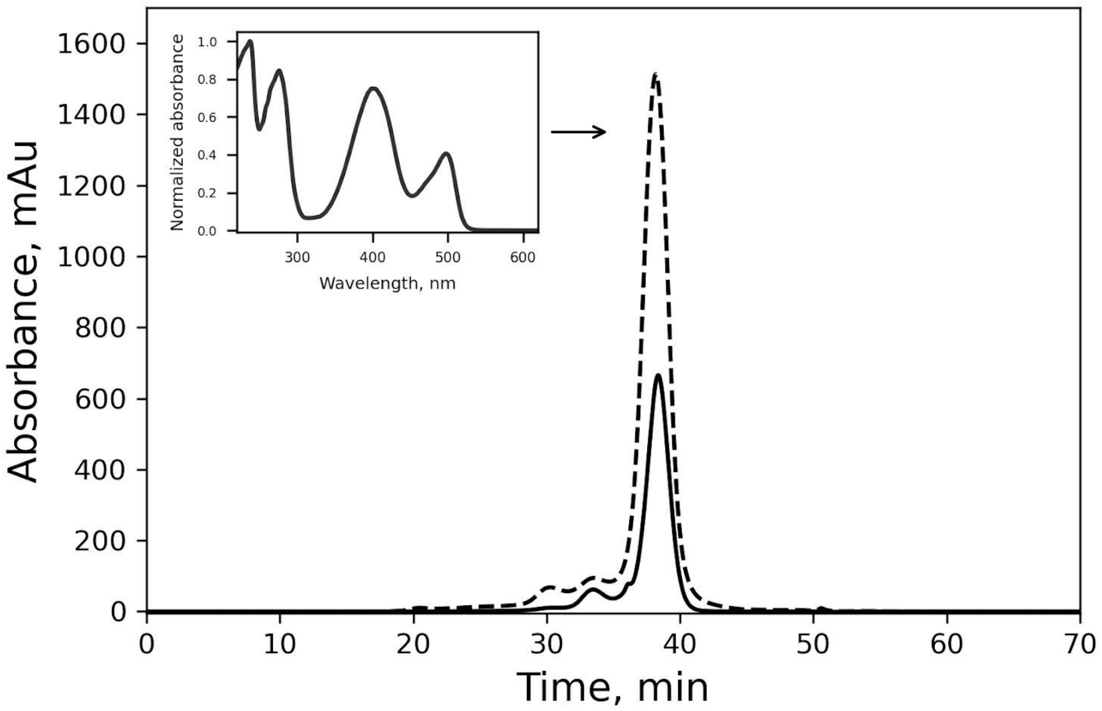

Supplementary Figure S10. Chromatographic profiles of GCaMP6s-BrUS batch 2 on Superdex 200 column monitored at wavelengths 280 nm (dashed line) and 488 nm (solid line). Inset: normalized absorption spectrum of GCaMP6s-BrUS.

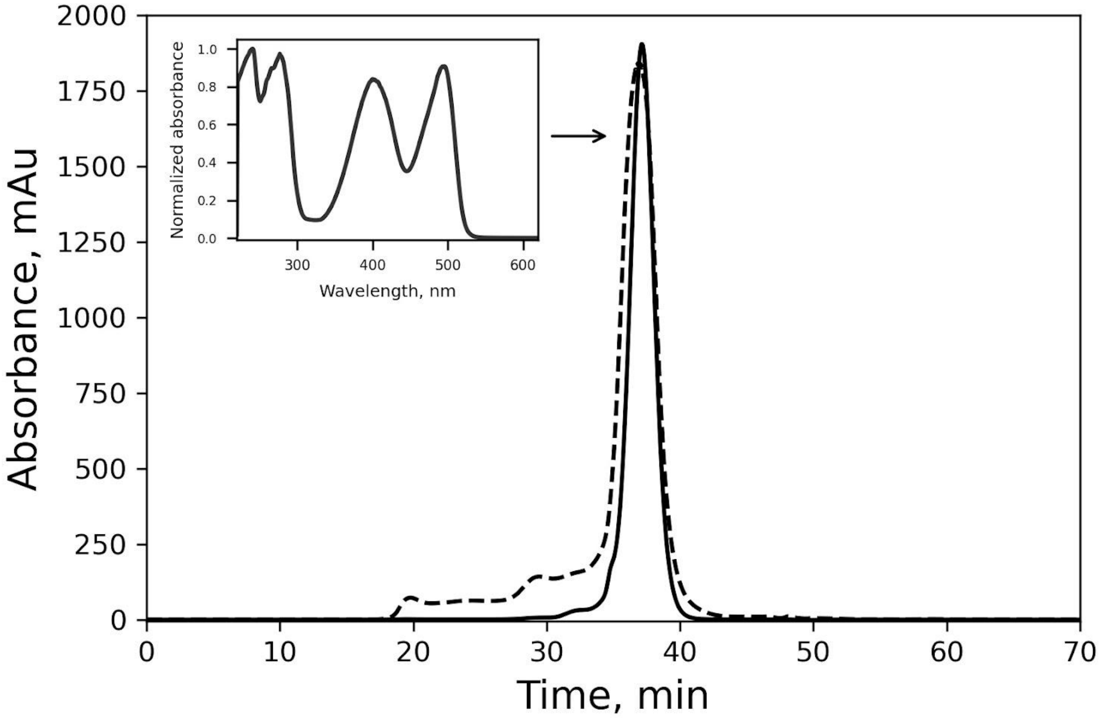

Supplementary Figure S11. Chromatographic profiles of GCaMP6s-BrUS-145 batch 1 on Superdex 200 column monitored at wavelengths 280 nm (dashed line) and 488 nm (solid line). Inset: normalized absorption spectrum of GCaMP6s-BrUS-145.

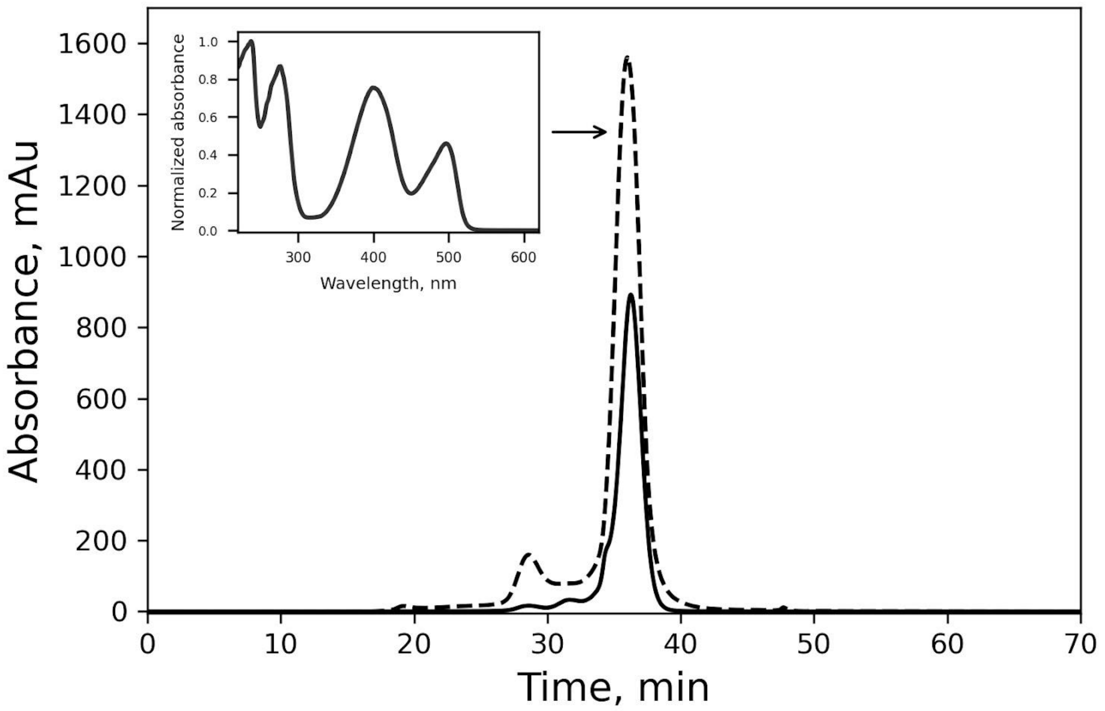

Supplementary Figure S12. Chromatographic profiles of GCaMP6s-BrUS-145 batch 2 on Superdex 200 column monitored at wavelengths 280 nm (dashed line) and 488 nm (solid line). Inset: normalized absorption spectrum of GCaMP6s-BrUS-145.

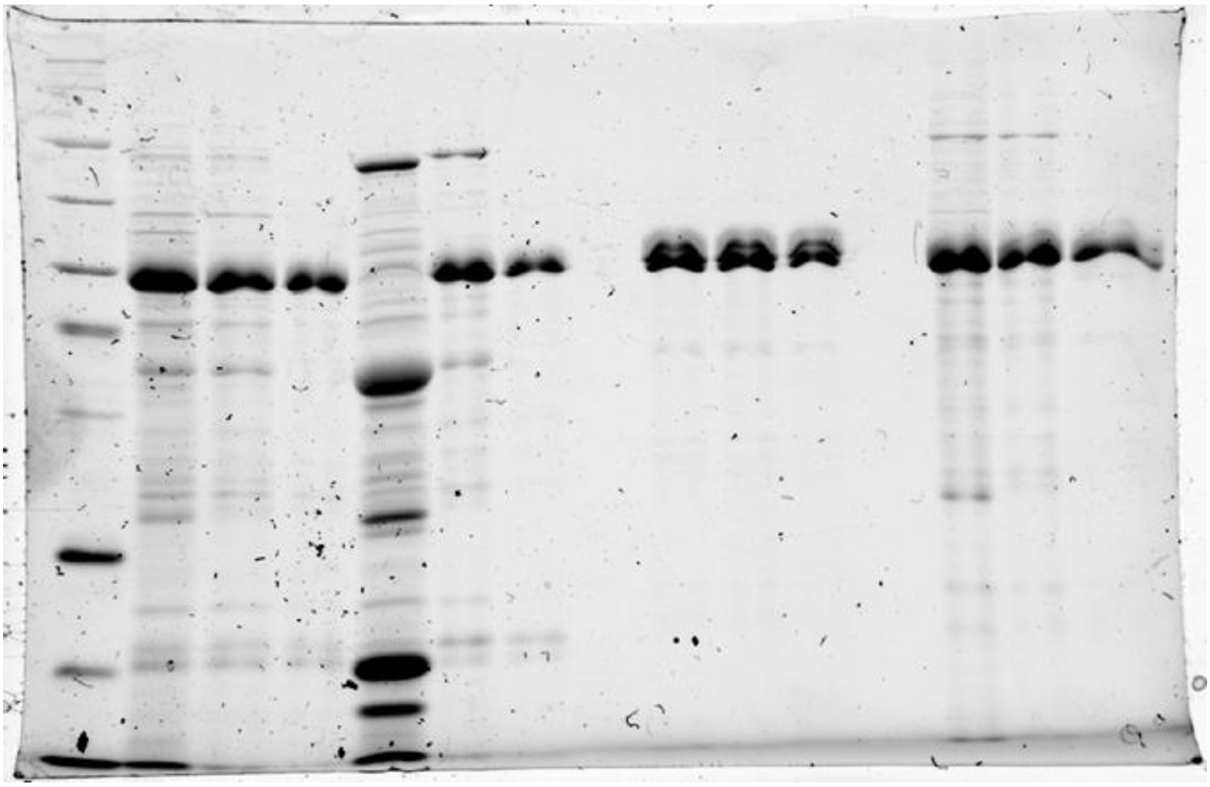

Supplementary Figure S13. SDS-PAGE analysis of expressed and purified proteins. Lanes: 1 – PageRuler Unstained Broad Range Protein Ladder, 2 - GCaMP6s-BrUS-145 batch 1 after immobilized metal affinity chromatography, 3 - GCaMP6s-BrUS-145 batch 1 after anion exchange chromatography, 4 - GCaMP6s-BrUS-145 batch 1 after size exclusion chromatography, 5 - GCaMP6s-BrUS-145 batch 2 after immobilized metal affinity chromatography, 6 - GCaMP6s-BrUS-145 batch 2 after anion exchange chromatography, 7 - GCaMP6s-BrUS-145 batch 2 after size exclusion chromatography, 8 - GCaMP6s-BrUS batch 1 after immobilized metal affinity chromatography, 9 - GCaMP6s-BrUS batch 1 after anion exchange chromatography, 10 - GCaMP6s-BrUS batch 1 after size exclusion chromatography, 11 - GCaMP6s-BrUS batch 2 after immobilized metal affinity chromatography, 12 - GCaMP6s-BrUS batch 2 after anion exchange chromatography, 13 - GCaMP6s-BrUS batch 2 after size exclusion chromatography.

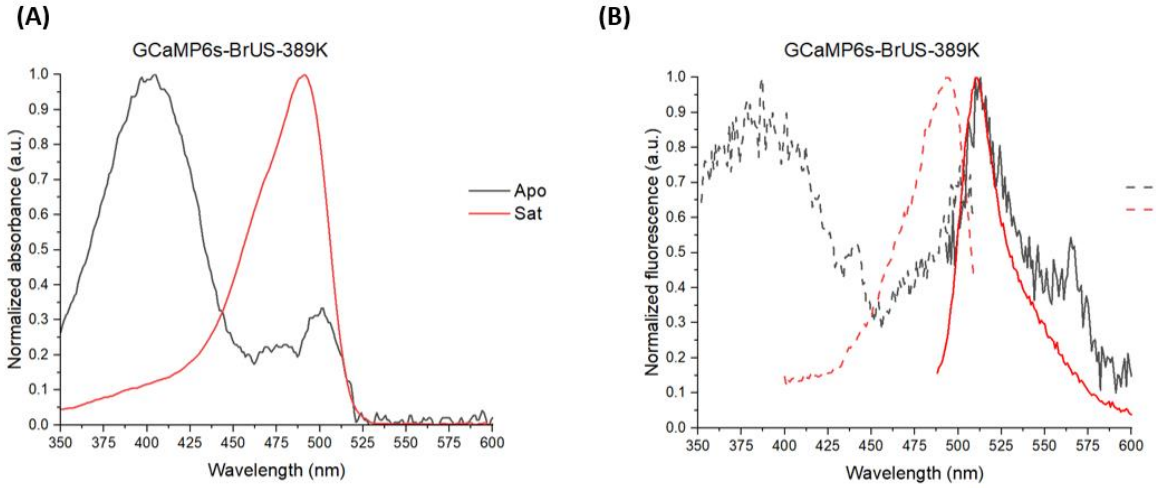

Supplementary Figure S14. Absorption (**A**) and fluorescence (**B**) spectra of the purified GCaMP6s-BrUS-389K protein in both its calcium-free (Apo) and calcium-saturated (Sat) forms. In the fluorescence graph, dashed lines show excitation spectra (λ_{em} = 525 nm), solid lines – emission spectra (λ_{ex} = 400 nm for Apo form, λ_{ex} = 475 nm for Sat form).

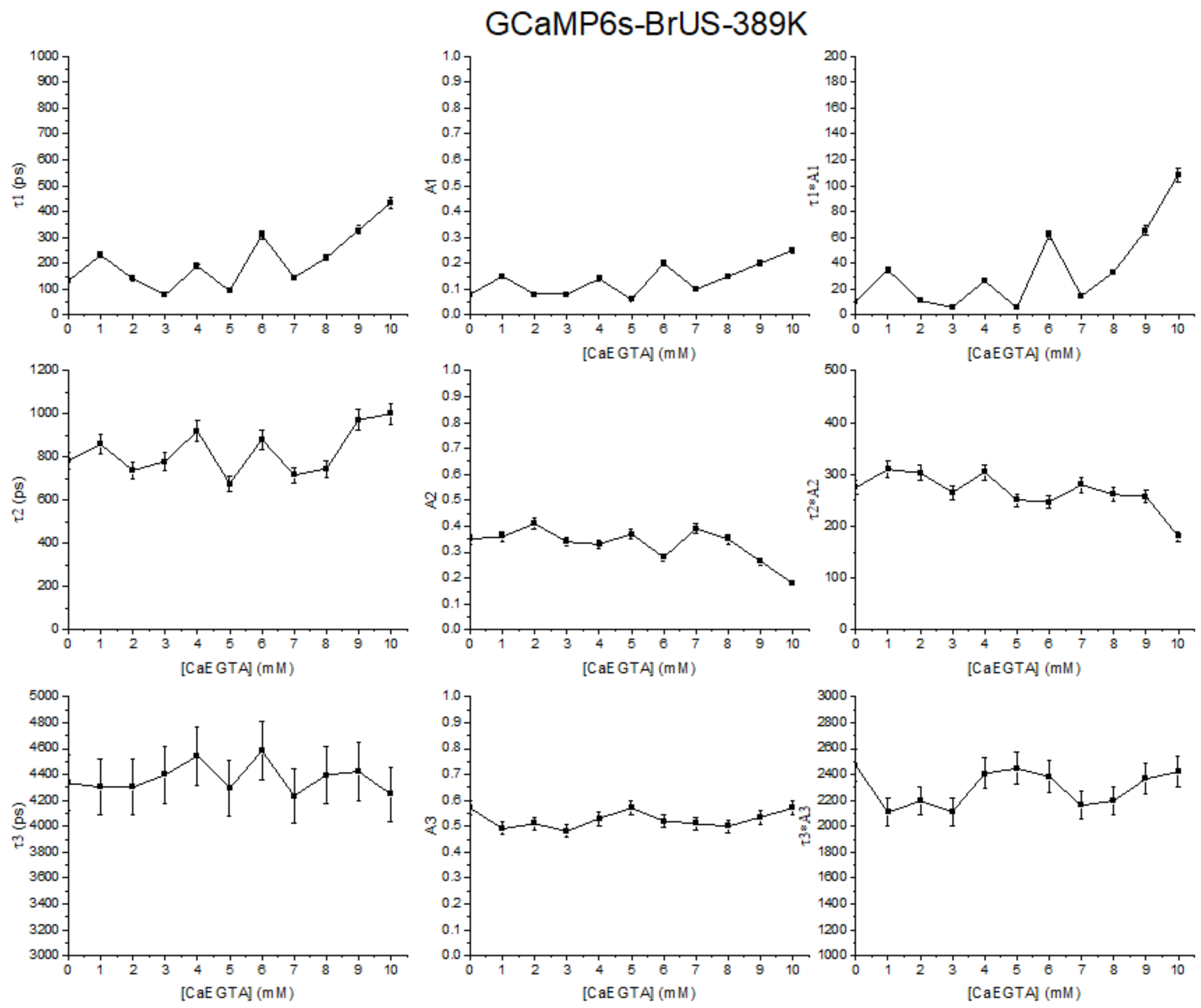

Supplementary Figure S15. Fluorescence decay kinetics of GCaMP6s-BrUS-389K fitted by a triexponential model, recorded at various calcium concentrations upon excitation with a 450 nm picosecond laser.

Supplementary Table S2. The assessment of brightness and calcium sensitivity of the indicator variants derived from the GCaMP6s-BrUS-R389K-based library after the second round of random mutagenesis.

Protein (amino acid substitutions)	Brightness[b]	Calcium sensitivity[c] (spectral domain)	Calcium sensitivity[d] (time domain)
R389K[a]/E398G	+	high	high
R389K/K259E/A330G	+	-	-
R389K/G260S/Q318P/K330M	+	low	-
R389K/L143V/G260S/F269Q	+	-	-
R389K/Q108G/V170G/K300Q/ Q317K/G355R/N357I	+	-	-

[a]R389K (in italics) is a site-directed structure-based mutation;

[b]Represented qualitatively: "+" indicates that the apparent brightness in bacterial colonies 24 h post-transformation was higher than that of the parental GCaMP6s-BrUS-389K;

[c]Represented qualitatively: "-" indicates there was no difference in the fluorescence intensity between Apo and Sat forms of purified protein; "low" – the ratio of fluorescence intensity between Apo and Sat forms was less than 1.5; "high" – the ratio was more than 5;

[d]Represented qualitatively: "-" indicates that the difference in fluorescence lifetime values between Apo and Sat forms of purified protein was less than 100 ps; "high" – the lifetime difference was more than 500 ps.

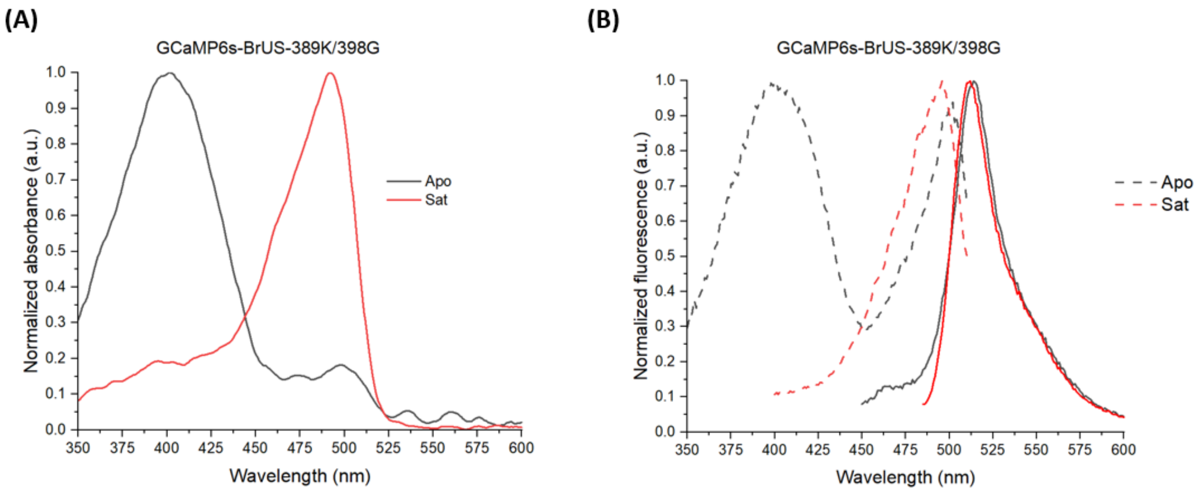

Supplementary Figure S16. Absorption **(A)** and fluorescence **(B)** spectra of the purified GCaMP6s-BrUS-389K/398G protein in both its calcium-free (Apo) and calcium-saturated (Sat) forms. In the fluorescence graph, dashed lines show excitation spectra (λ_{em} = 525 nm), solid lines – emission spectra (λ_{ex} = 400 nm for Apo form, λ_{ex} = 475 nm for Sat form).

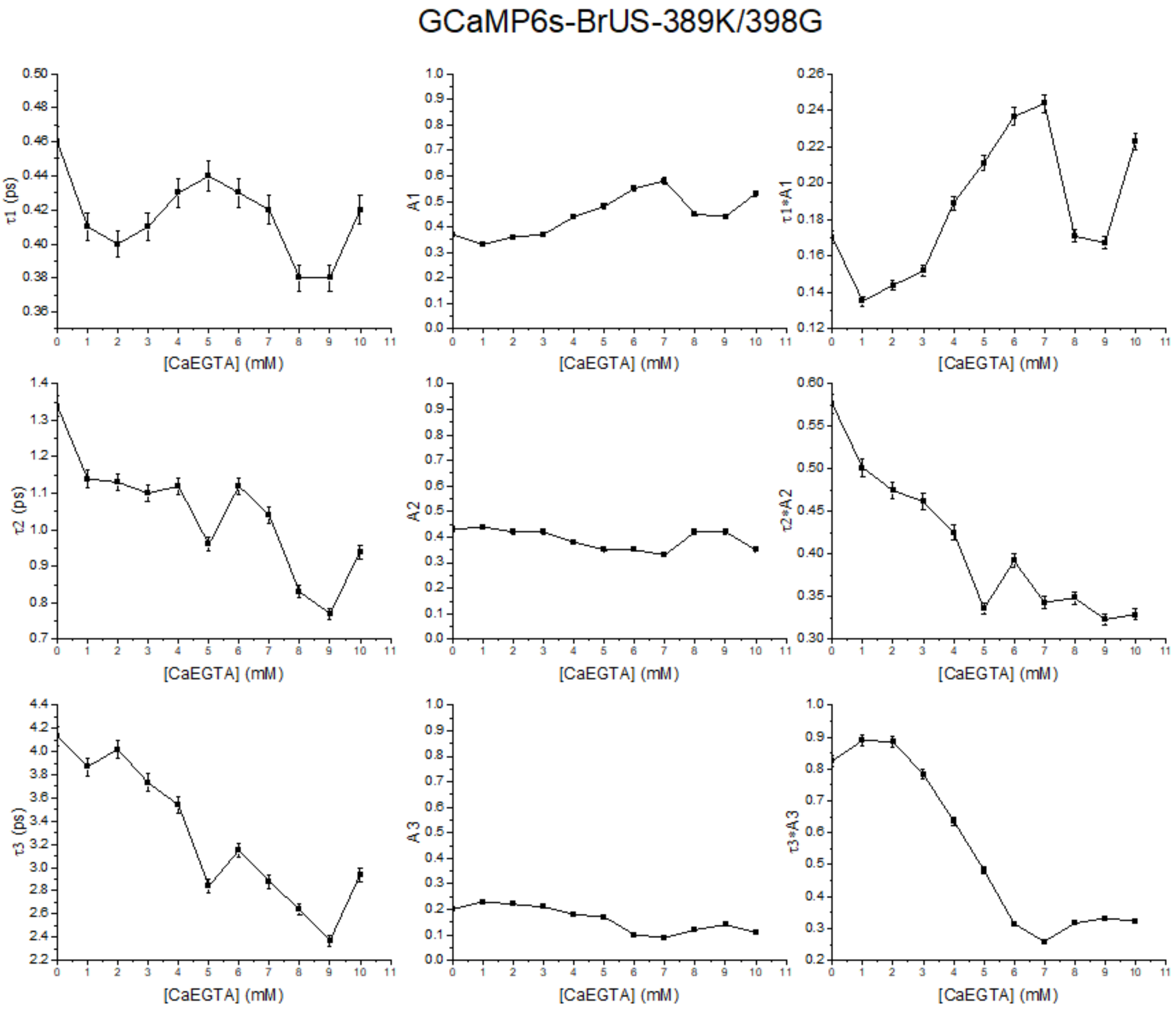

Supplementary Figure S17. Fluorescence decay kinetics of GCaMP-BrUSLEE-389K/398G fitted by a triexponential model, recorded at various calcium concentrations upon excitation with a 450 nm picosecond laser.

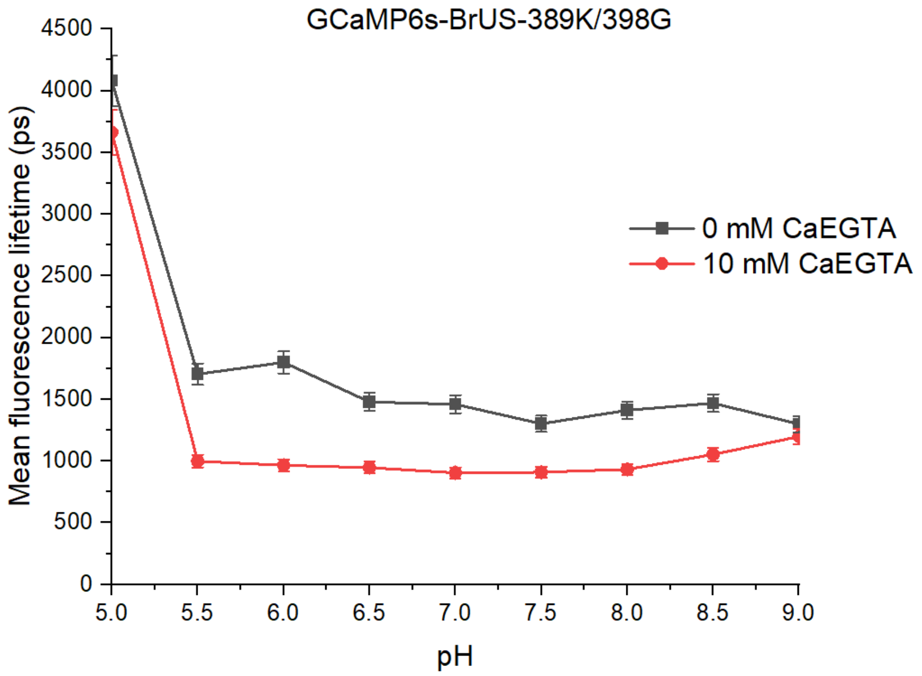

Supplementary Figure S18. Sensitivity of the GCaMP-BrUS-389K/398G mean fluorescence lifetime to pH in the calcium-bound and -unbound states in vitro. Standard errors of the mean (S.E.M.) are shown for each data point (n = 3).

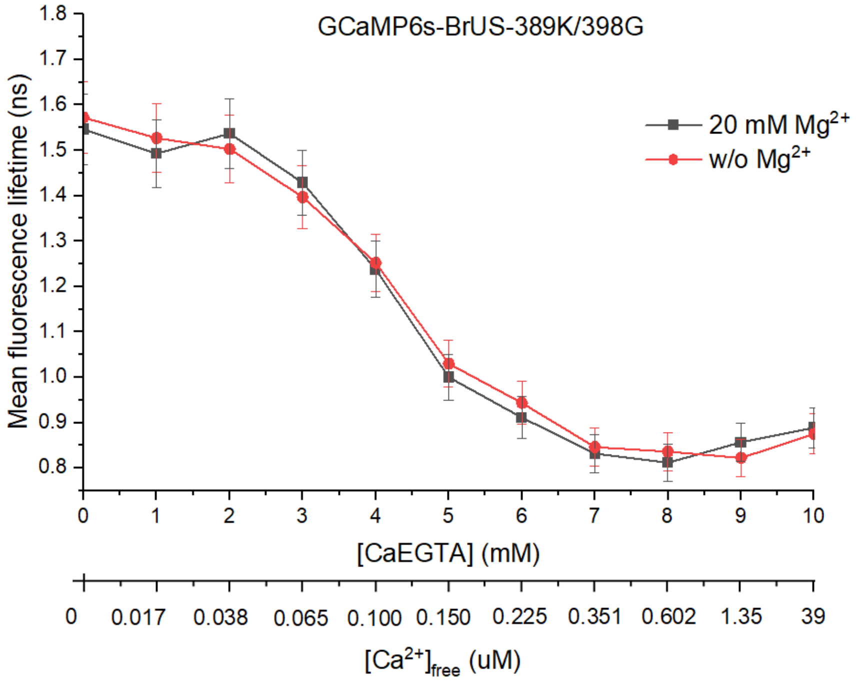

Supplementary Figure S19. Sensitivity of the calcium-dependent fluorescence lifetime changes of GCaMP-BrUS-389K/398G to the Mg^{2+} cation. Standard errors of the mean (S.E.M.) are shown for each data point (n = 3).

Supplementary Table S3. Data collection and refinement statistics.

	**GCaMP6s-BrUS**	**GCaMP6s-BrUS-145**
**Data collection**		
Diffraction source	LNLS (Manaca beamline)	XtaLAB Synergy-S, Rigaku
Wavelength (Å)	0.9772	1.5418
Detector	Dectris Eiger 2	HyPix-6000HE
Temperature (K)	100	100
Space group	P41212	P41212
a, b, c (Å)	121.54 121.54 97.90	118.29 118.29 98.70
Resolution range (Å)	47.52-2.65 (2.78-2.65)	23.01-2.05 (2.11-2.05)
Completeness (%)	99.3 (99.8)	99.9 (100.0)
Redundancy	7.2	24.8
⟨I/σ(I)⟩	9.1 (1.5)	17.2 (1.9)
Rmeas (%)	19.7 (139.0)	18.8 (178.8)
CC1/2 (%)	99.4 (49.1)	99.9 (83.0)
**Refinement**		
Final Rcryst (%)	18.2	21.1
Final Rfree (%)	23.7	25.1
No. of non-H atoms		
Protein	3117	3117
Chromophore	19	19

Calcium	4	4
Other	-	29
Water	90	286
R.m.s. deviations		
Bonds (Å)	0.01	0.02
Angles (°)	2.94	2.61
Average B factors (Å2)		
Protein	55.2	35.3
Chromophore	40.5	23.1
Calcium	52.1	30.9
Other	-	21.6
Water	41.2	31.4
**PDB ID**	**9K8W**	**9K8X**
